# Supplementary material for: Overcoming Computational Bottlenecks in Quantum Hydrodynamics: A Volume‐Based Integral Formalism for Spherical Nanoparticles
Source: Nanophotonics. 2026 Jun 30;15(13):e70179. doi: 10.1002/nap2.70179 (PMC13642565; doi:10.1002/nap2.70179)
Supplement: Supplementary file 1 [file NAP2-15-e70179-s001.pdf]

Supporting Information: Overcoming Computational Bottlenecks in Quantum Hydrodynamics: A Volume-Based Integral Formalism for Spherical Nanoparticles

Christos Mystilidis Christos Tserkezis N. Asger Mortensen Guy A. E. Vandenbosch Xuezhi Zheng**

Dr. Christos Mystilidis
Email address: chrmys@mci.sdu.dk
Address: POLIMA—Center for Polariton-driven Light-—Matter Interactions, University of Southern Denmark, DK-5230 Odense, Denmark. School of Electrical and Computer Engineering, National Technical University of Athens, 9 Iroon Polytechniou Street GR 157-73 Zografou, Athens, Greece.

Prof. Christos Tserkezis
Address: POLIMA—Center for Polariton-driven Light-—Matter Interactions, University of Southern Denmark, DK-5230 Odense, Denmark. D-IAS: Danish Institute for Advanced Study, University of Southern Denmark, Campusvej 55, DK-5230 Odense M, Denmark.

Prof. Guy A. E. Vandenbosch
Address: WaveCoRE Division, Department of Electrical Engineering (ESAT), KU Leuven, Kasteelpark Arenberg 10, box 2444, Leuven, B-3001, Belgium.

Prof. N. Asger Mortensen
Address: POLIMA—-Center for Polariton-driven Light-—Matter Interactions, University of Southern Denmark, DK-5230 Odense, Denmark. D-IAS: Danish Institute for Advanced Study, University of Southern Denmark, Campusvej 55, DK-5230 Odense M, Denmark.

Prof. Xuezhi Zheng
Email Address: xzheng@nuaa.edu.cn
Address: College of Electronics and Information Engineering, Nanjing University of Aeronautics and Astronautics, Nanjing, China. National Key Laboratory of Microwave Photonics, Nanjing University of Aeronautics and Astronautics, Nanjing, China.

S1 On the tail of the ground-state density

As discussed in the main text, we model the monotonous decay of the ground-state density across the interface using a hyperbolic tangent, in particular [S1]

$$n_0(r) = \frac{n_0}{2}\left[1 - \tanh\left(\frac{r-R}{a_n}\right)\right], \tag{S1}$$

where the symbols have been defined in the main text. In our MATLAB implementation we noticed that such a function displays a monotonic rather than strictly monotonic behavior for $(r-R)/a_n \gg 1$. This may lead to abrupt drops to zero and as such increasing the computational domain (by increasing the spill-out allowance s) becomes prohibitive. To mitigate this problem, we calculate the correct asymptotic decay of Equation (S1) and enforce it after a sufficient distance from the jellium edge. The final ground-state density used is

$$n_0(r) = \begin{cases} \dfrac{n_0}{2}\left[1 - \tanh\left(\dfrac{r-R}{a_n}\right)\right], & \dfrac{r-R}{a_n} \leq 3, \\ n_0 e^{-2\left(\dfrac{r-R}{a_n}\right)}, & \dfrac{r-R}{a_n} > 3. \end{cases} \tag{S2}$$

S2 On a_n

The parameter a_n is the most critical parameter of the model ground-state density, reflecting its decay across the interface. Its value determines how much the electron density overcomes the boundary and spills in the electromagnetic background; it is, as such, a measure of electron spill-out, and the optical response of the nanospheres involved is influenced decisively by it. At the same time, irrespective of the model used, it must reflect the physical reality of the material at hand, in this case sodium (Na). In order to acquire a reasonable value of a_n, we follow [S2]. There, the decay rate κ (notice that a different model is used) is extracted by comparison with the asymptotic decay predicted by full Density Functional Theory (DFT), ending up with $\kappa = 1.05/a_0$, where a_0 is the Bohr radius, independent of the size of the nanoparticle (and thus effectively bypassing the need for full calculations at the ground state either using DFT or the truly self-consistent Self-Consistent Hydrodynamic Drude Model—SC-HDM).

Here, we match our model with the one used in [S2] (which in turn is matched to DFT), requiring them to be equivalent (that is, to have equivalent decays) in the limits $r - R \gg a_n$ and $r - R \gg \kappa^{-1}$, respectively. This matching gives[1]

$$a_n = \frac{2}{\kappa}, \tag{S3}$$

which we use throughout. The influence of a_n in the electron density profile, as well as the agreement between the two models discussed using in the first a_n as above, and the second $\kappa = 1.05/a_0$, are shown in Figure S1.

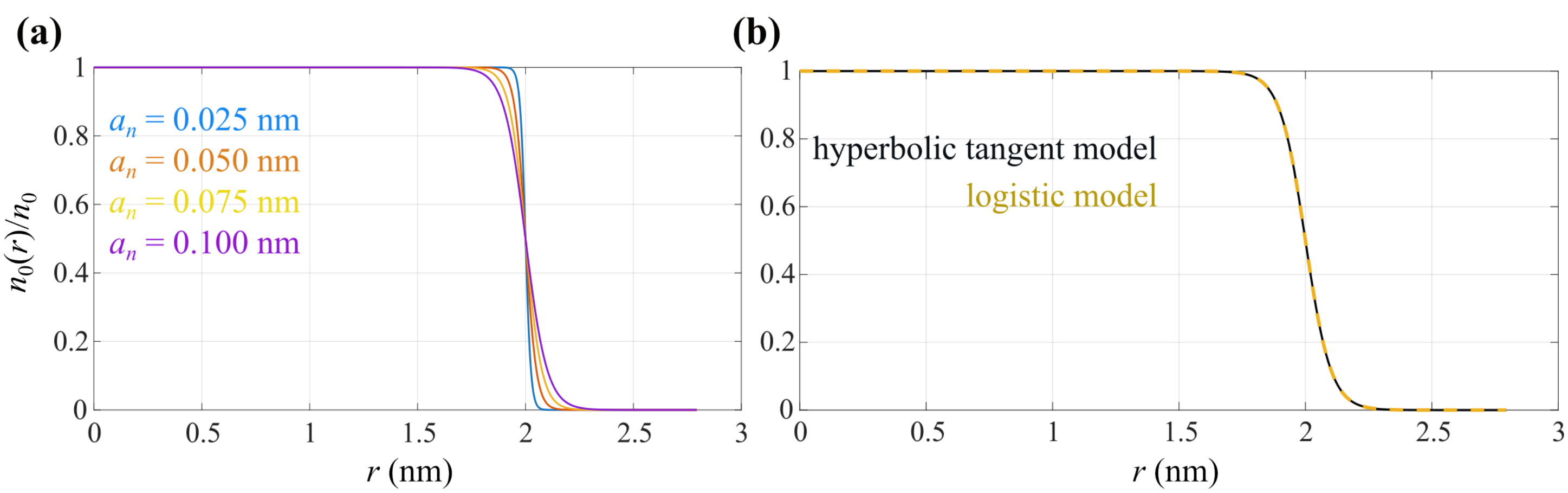

Figure S1: (a) Influence of the decay rate a_n on the density profile. As we increase a_n the nanoparticle gets diffused deeper in the background. The interface becomes smoother. (b) Agreement between Equation (S2) and the logistic model of [S2] under the conditions of Equation (S3).

S3 On $G[n]$

In this section we detail the various contributions to the internal energy functional depending on the level of sophistication of the material response. Such expressions exist in the literature (see for example [S2], [S3]) and we repeat them here for bookkeeping purposes.

For the classical LRA the situation is simply $G[n] = 0$. At the Hydrodynamic-Drude-Model (HDM) level of description, the Thomas-Fermi kinetic energy suffices. This is[2] [S2], [S4]

$$T_{\mathrm{TF}}[n] = (E_h a_0^2) C_{\mathrm{TF}} \int n(\mathbf{r})^{\frac{5}{3}} d\mathbf{r}, \quad \left(\frac{\delta T_{\mathrm{TF}}[n]}{\delta n}\right)_1 = \left(E_h a_0^2\right) \frac{10}{9} C_{\mathrm{TF}} n_0^{-\frac{1}{3}}(\mathbf{r}) n_1(\mathbf{r}) = \gamma_{\mathrm{TF}}(\mathbf{r}) n_1(\mathbf{r}), \tag{S4}$$

[1] Note a slight difference between the two approaches: in our case, the bulk ground-state density n_0 is imposed as a parameter. In [S2], the amplitude of $n_0(r)$ is such that it reproduces the number of electrons in line with DFT, and reflecting the physical reality. Our lens here, on the other hand, reflects that SC-HDM, even though advanced and sophisticated, remains a *continuum* model.

[2] In the following, we give both the functional and its functional derivative (the subscript 1 clarifies that $\left(\frac{\delta G[n]}{\delta n}\right)_1 = \left(\frac{\delta G[n]}{\delta n}\right)_{n_0+n_1} - \left(\frac{\delta G[n]}{\delta n}\right)_{n_0}$ [S3], taken to the first order of n_1; in other words, this is the variation of the first functional derivative.

where E_h is the Hartree energy, $C_{\mathrm{TF}} = \frac{3}{10}(3\pi^2)^{2/3}$ the Thomas-Fermi factor, and $\gamma_{\mathrm{TF}}(\mathbf{r})$ a short-hand notation we introduce in order to clarify the dependence of the Thomas-Fermi (but also others, see below) functional derivative on the induced charge density (and possibly its derivatives).

At the SC-HDM level, the von Weizsäcker correction is added—this enables the introduction of a spatially varying ground-state density—and is [S2], [S3]

$$T_{\mathrm{vW}}[n] = (E_h a_0^2)\frac{1}{8}\int \frac{|\nabla n(\mathbf{r})|^2}{n(\mathbf{r})} d\mathbf{r},$$
$$\left(\frac{\delta T_{\mathrm{vW}}[n]}{\delta n}\right)_1 = \left(E_h a_0^2\right)\frac{1}{4}\left[\frac{\nabla^2 n_0(\mathbf{r})}{n_0^2(\mathbf{r})}n_1(\mathbf{r}) - \frac{|\nabla n_0(\mathbf{r})|^2}{n_0^3(\mathbf{r})}n_1(\mathbf{r}) + \frac{\nabla n_0(\mathbf{r})}{n_0^2(\mathbf{r})}\cdot\nabla n_1(\mathbf{r}) - \frac{1}{n_0(\mathbf{r})}\nabla^2 n_1(\mathbf{r})\right], \tag{S5}$$

or in a more compact manner

$$T_{\mathrm{vW}}[n] = (E_h a_0^2)\frac{1}{8}\int \frac{|\nabla n(\mathbf{r})|^2}{n(\mathbf{r})} d\mathbf{r}, \quad \left(\frac{\delta T_{\mathrm{vW}}[n]}{\delta n}\right)_1 = \gamma_{\mathrm{vW},1}(\mathbf{r})n_1(\mathbf{r}) + \boldsymbol{\gamma}_{\mathrm{vW},2}(\mathbf{r})\cdot\nabla n_1(\mathbf{r}) + \gamma_{\mathrm{vW},3}(\mathbf{r})\nabla^2 n_1(\mathbf{r}). \tag{S6}$$

Note that we use the weight $1/\eta$ when introducing the contribution above in our formalism and absorb it in the material functions γ_{vW}.

We next proceed with the Local Density Approximation (LDA) of the Exchange-Correlation (XC) energy, which is [S5]

$$E_{\mathrm{XC}}[n] = E_{\mathrm{X}}[n] + E_{\mathrm{C}}[n] = -(E_h a_0)C_{\mathrm{X}}\int n^{\frac{4}{3}}(\mathbf{r})d\mathbf{r} + E_h\int \varepsilon_{\mathrm{C}}[n]n(\mathbf{r})d\mathbf{r}, \tag{S7}$$

where $C_{\mathrm{X}} = \frac{3}{4}(3/\pi)^{\frac{1}{3}}$ is the X factor and ε_{C} is the average C energy per electron, given by [S6]

$$\varepsilon_{\mathrm{C}}[n] = \begin{cases} a\ln r_s + b + c r_s\ln r_s + d r_s, & r_s < 1,\\ \dfrac{\alpha}{1+\beta_1\sqrt{r_s}+\beta_2 r_s}, & r_s \geq 1,\end{cases} \tag{S8}$$

where $a_0 r_s = (3/(4\pi n(\mathbf{r})))^{1/3}$ is the normalized Wigner-Seitz radius, and the constants $a, b, c, d, \alpha, \beta_1, \beta_2$ are real numbers given in the original work [S6]. As for the functional derivative [S2]

$$\left(\frac{\delta E_{\mathrm{XC}}[n]}{\delta n}\right)_1 = -\left(E_h a_0\right)\frac{4}{9}C_{\mathrm{X}} n_0^{-\frac{2}{3}}(\mathbf{r})n_1(\mathbf{r}) + (E_h a_0^3)\mu_{\mathrm{C}}'[n_0]n_1(\mathbf{r}) = \gamma_{\mathrm{XC}}(\mathbf{r})n_1(\mathbf{r}), \tag{S9}$$

where [S2]

$$\mu_{\mathrm{C}}'[n_0(\mathbf{r})] = \begin{cases} -\frac{4\pi}{9}\left(a + \frac{1}{3}(1+2\ln r_s)c r_s + \frac{2}{3}d r_s\right) r_s^3, & r_s < 1,\\ \dfrac{\alpha\pi}{27}\dfrac{5\beta_1\sqrt{r_s} + (7\beta_1^2 + 8\beta_2)r_s + 21\beta_1\beta_2 r_s^{\frac{3}{2}} + 16\beta_2^2 r_s^2}{(1+\beta_1\sqrt{r_s}+\beta_2 r_s)^3} r_s^3, & r_s \geq 1.\end{cases} \tag{S10}$$

S4 On the plane-wave excitation

The method can readily be generalized to any conventional excitation used in the literature (that is, dipole and electron-beam excitations), however the current implementation deals exclusively with a plane-wave excitation.

The treatment of the plane wave follows Mie-theory considerations of the field (see for example [S7]) and requires the translation of the plane wave to a superposition of spherical waves, a well-documented procedure [S8]. Here we give the final expressions of the relevant Mie coefficients. In more detail, the incident field is expanded as

$$\mathbf{E}_{\mathrm{inc}}(\mathbf{r}) = \sum_{l=1}^{\infty}\sum_{m=-l}^{l} a_{lm}^{(\mathrm{inc})}\mathbf{M}_{lm}^{(1)}(\mathbf{r}) + b_{lm}^{(\mathrm{inc})}\mathbf{N}_{lm}^{(1)}(\mathbf{r}), \tag{S11}$$

where the spherical vector wave functions $\mathbf{M}_{lm}^{(1)}(\mathbf{r})$ and $\mathbf{N}_{lm}^{(1)}(\mathbf{r})$ are understood to contain spherical Bessel functions (for more details see [S7] and the textbook [S9]), and $a_{lm}^{(\mathrm{inc})}$ and $b_{lm}^{(\mathrm{inc})}$ are the Mie coefficients. Since the space around the spherical scatterer is purely local (vacuum), we use exclusively transverse wave functions in this representation. Notice that a very small number of terms in Equation (S11) is required for an accurate representation of the plane wave (and due to the homogeneity thereof). The Mie coefficients are

$$a_{lm}^{(\mathrm{inc})} = 4\pi \frac{i^l}{l(l+1)} (-1)^m \mathbf{X}_{l,-m}(\theta_{\mathrm{inc}}, \phi_{\mathrm{inc}}) \cdot \mathbf{E}_0(\mathbf{k}), \quad \text{and} \quad b_{lm}^{(\mathrm{inc})} = 4\pi \frac{i^{l+1}}{l(l+1)} (-1)^{m+1} \mathbf{Z}_{l,-m}(\theta_{\mathrm{inc}}, \phi_{\mathrm{inc}}) \cdot \mathbf{E}_0(\mathbf{k}), \tag{S12}$$

where $\mathbf{X}$ and $\mathbf{Z}$ have been defined in the main text and $(\theta_{\mathrm{inc}}, \phi_{\mathrm{inc}})$ are the elevation and azimuth incident angles. We further clarify that we always select the amplitude to be $|\mathbf{E}_0(\mathbf{k})| = 1$ V/m, which is merely a scaling constant.

The polarization of the incident electric field is defined by $\mathbf{E}_0(\mathbf{k})$. For the popular Transverse Electric (TE) and Transverse Magnetic (TM) cases, the plane wave is completely described by the *planar* vector wave functions, i.e., a TE wave contains only $\mathbf{M}$ and a TM wave contains only $\mathbf{N}$. In the context of spherical geometries (and spherical vector wave functions), it translates to $\hat{\boldsymbol{\phi}}$ (for TE waves) and $\hat{\boldsymbol{\theta}}$ (for TM waves).

S5 Arbitrary construction

In this Section, we derive the linear system, from the initial working equation (see Equation (9) of the main text). The construction aspires to be educational, demonstrating the introduction of the auxiliary variable, the basis functions, and the projection onto testing functions.

We recall that

$$-\frac{1}{e}\nabla\left(\frac{\delta G[n]}{\delta n}\right)_1 + \frac{1}{\varepsilon_0 \chi(\mathbf{r};\omega)}\mathbf{P}(\mathbf{r}) - \omega^2\mu_0 \int_{V'} \overline{\mathbf{G}}(\mathbf{r},\mathbf{r}') \cdot \mathbf{P}(\mathbf{r}')d\mathbf{r}' = \mathbf{E}_{\mathrm{inc}}(\mathbf{r}), \tag{S13}$$

where the symbols have been defined in the main text. This is the working equation of our algorithm. We notice that

$$\frac{\delta G[n]}{\delta n} = \frac{\delta G[n;\nabla n;\nabla^2 n]}{\delta n}, \tag{S14}$$

following from Equation (3) of the main text and the level of material description for SC-HDM[3]. To relax the differentiability requirements for our expansion basis, we introduce an auxiliary variable

$$\mathbf{F}(\mathbf{r}) = e\nabla n_1(\mathbf{r}), \quad \nabla \cdot \mathbf{F}(\mathbf{r}) = e\nabla^2 n_1(\mathbf{r}), \tag{S15}$$

or

$$\mathbf{F}(\mathbf{r}) = \nabla(\nabla \cdot \mathbf{P}(\mathbf{r})), \tag{S16}$$

where we used $en_1(\mathbf{r}) = \nabla \cdot \mathbf{P}(\mathbf{r})$. We perform testing of Equation (S13) by an arbitrary function $\tilde{\mathbf{P}}(\mathbf{r})$, leading to

$$\begin{aligned} &-\frac{1}{e}\int_V \tilde{\mathbf{P}}(\mathbf{r}) \cdot \nabla\left(\frac{\delta G[n]}{\delta n}\right)_1 d\mathbf{r} + \frac{1}{\varepsilon_0}\int_V \tilde{\mathbf{P}}(\mathbf{r}) \cdot \frac{1}{\chi(\mathbf{r};\omega)}\mathbf{P}(\mathbf{r})d\mathbf{r} \\ &-\omega^2\mu_0 \int_V \tilde{\mathbf{P}}(\mathbf{r}) \cdot \int_{V'} \overline{\mathbf{G}}(\mathbf{r},\mathbf{r}') \cdot \mathbf{P}(\mathbf{r}')d\mathbf{r}'d\mathbf{r} = \int_V \tilde{\mathbf{P}}(\mathbf{r}) \cdot \mathbf{E}_{\mathrm{inc}}(\mathbf{r})d\mathbf{r}, \end{aligned} \tag{S17}$$

with the understanding that the first term above contains $\mathbf{F}(\mathbf{r})$ and $\nabla \cdot \mathbf{F}(\mathbf{r})$ (in addition to $\nabla \cdot \mathbf{P}(\mathbf{r})$; notice that the same order of derivative is distributed to both unknown functions). We use integration by parts on the first term and *assume* that the boundary contributions vanish; this is a critical part of the derivation and is related to how the boundary conditions are introduced in our formalism. For more details see the relevant Section S11. The

[3] Note that this dependence on the derivatives of n comes in our description solely from the von Weizsäcker kinetic term.

procedure leads to[4]

$$\frac{1}{e}\int_V \nabla\cdot\tilde{\mathbf{P}}(\mathbf{r})\cdot\left(\frac{\delta G[n]}{\delta n}\right)_1 d\mathbf{r} + \frac{1}{\varepsilon_0}\int_V \tilde{\mathbf{P}}(\mathbf{r})\cdot\frac{1}{\chi(\mathbf{r};\omega)}\mathbf{P}(\mathbf{r})d\mathbf{r} \\ -\omega^2\mu_0\int_V \tilde{\mathbf{P}}(\mathbf{r})\cdot\int_{V'}\overline{\mathbf{G}}(\mathbf{r},\mathbf{r}')\cdot\mathbf{P}(\mathbf{r}')d\mathbf{r}'d\mathbf{r} = \int_V \tilde{\mathbf{P}}(\mathbf{r})\cdot\mathbf{E}_{\text{inc}}(\mathbf{r})d\mathbf{r}. \tag{S18}$$

We use Equation (3) of the main text to get

$$\frac{1}{e}\int_V \nabla\cdot\tilde{\mathbf{P}}(\mathbf{r})\left[\gamma_{\text{TF}}(\mathbf{r})+\gamma_{\text{XC}}(\mathbf{r})+\gamma_{\text{vW},1}(\mathbf{r})\right]n_1(\mathbf{r})d\mathbf{r} + \frac{1}{e}\int_V \nabla\cdot\tilde{\mathbf{P}}(\mathbf{r})\boldsymbol{\gamma}_{\text{vW},2}(\mathbf{r})\cdot\nabla n_1(\mathbf{r})d\mathbf{r} + \\ \frac{1}{e}\int_V \nabla\cdot\tilde{\mathbf{P}}(\mathbf{r})\gamma_{\text{vW},3}(\mathbf{r})\nabla^2 n_1(\mathbf{r})d\mathbf{r} + \frac{1}{\varepsilon_0}\int_V \tilde{\mathbf{P}}(\mathbf{r})\cdot\frac{1}{\chi(\mathbf{r};\omega)}\mathbf{P}(\mathbf{r})d\mathbf{r} - \\ \omega^2\mu_0\int_V \tilde{\mathbf{P}}(\mathbf{r})\cdot\int_{V'}\overline{\mathbf{G}}(\mathbf{r},\mathbf{r}')\cdot\mathbf{P}(\mathbf{r}')d\mathbf{r}'d\mathbf{r} = \int_V \tilde{\mathbf{P}}(\mathbf{r})\cdot\mathbf{E}_{\text{inc}}(\mathbf{r})d\mathbf{r}, \tag{S19}$$

and eventually

$$\frac{1}{e^2}\int_V \nabla\cdot\tilde{\mathbf{P}}(\mathbf{r})\left[\gamma_{\text{TF}}(\mathbf{r})+\gamma_{\text{XC}}(\mathbf{r})+\gamma_{\text{vW},1}(\mathbf{r})\right]\nabla\cdot\mathbf{P}(\mathbf{r})d\mathbf{r} + \frac{1}{e^2}\int_V \nabla\cdot\tilde{\mathbf{P}}(\mathbf{r})\boldsymbol{\gamma}_{\text{vW},2}(\mathbf{r})\cdot\mathbf{F}(\mathbf{r})d\mathbf{r} + \\ \frac{1}{e^2}\int_V \nabla\cdot\tilde{\mathbf{P}}(\mathbf{r})\gamma_{\text{vW},3}(\mathbf{r})\nabla\cdot\mathbf{F}(\mathbf{r})d\mathbf{r} + \frac{1}{\varepsilon_0}\int_V \tilde{\mathbf{P}}(\mathbf{r})\cdot\frac{1}{\chi(\mathbf{r};\omega)}\mathbf{P}(\mathbf{r})d\mathbf{r} - \\ \omega^2\mu_0\int_V \tilde{\mathbf{P}}(\mathbf{r})\cdot\int_{V'}\overline{\mathbf{G}}(\mathbf{r},\mathbf{r}')\cdot\mathbf{P}(\mathbf{r}')d\mathbf{r}'d\mathbf{r} = \int_V \tilde{\mathbf{P}}(\mathbf{r})\cdot\mathbf{E}_{\text{inc}}(\mathbf{r})d\mathbf{r}. \tag{S20}$$

To make the system (in principle) solvable, we employ the auxiliary Equation (S16) and perform testing with another arbitrary function $\tilde{\mathbf{F}}(\mathbf{r})$

$$\int_V \tilde{\mathbf{F}}(\mathbf{r})\cdot\mathbf{F}(\mathbf{r})d\mathbf{r} - \int_V \tilde{\mathbf{F}}(\mathbf{r})\cdot\nabla(\nabla\cdot\mathbf{P}(\mathbf{r}))d\mathbf{r} = 0, \tag{S21}$$

and then we use integration by parts on the second term and assume vanishing of the boundary contributions,

$$\int_V \tilde{\mathbf{F}}(\mathbf{r})\cdot\mathbf{F}(\mathbf{r})d\mathbf{r} + \int_V \nabla\cdot\tilde{\mathbf{F}}(\mathbf{r})\nabla\cdot\mathbf{P}(\mathbf{r})d\mathbf{r} = 0. \tag{S22}$$

The system of Equations (S20) and (S22) lies at the heart of our method; comparison with Equation (13) of the main text is now straightforward. In the next we will elaborate on the contributions of each term separately[5].

S6 Elements, nodes, and indexing system

In the main text, we presented a discretization based on the number of basis functions used: The unknown variable (induced free-electron polarization density $\mathbf{P}(\mathbf{r})$) was expanded in a number of appropriate basis functions. Here we introduce a different (but equivalent) discretization scheme that better reflects our implementation.

We introduce the notions *elements* and *nodes*. Elements are the geometric unit cells on which the basis functions (we recall a product of polynomials for the radial part and vector spherical harmonics for the angular part) are defined. In 3D, the elements are tiny spherical shells (except for the first one, which is a spherical core), see Figure S2 (though we will see that the situation is simplified tremendously after significant analytic effort).

In each element, more than one basis function may reside. This is not necessary, but a choice following [S10]. The number of basis functions depends on the order of the polynomials we use to represent the radial variation.

[4]Notice that the procedure we follow here, and later for the auxiliary equation, by redistributing the derivatives from the unknown variable to the testing functions, is tantamount to deriving the weak form of Equation (S13).

[5]Note that in the construction we presented here, the testing functions are treated as having compact support; this is used for notational simplicity and is not the case for our solution strategy.

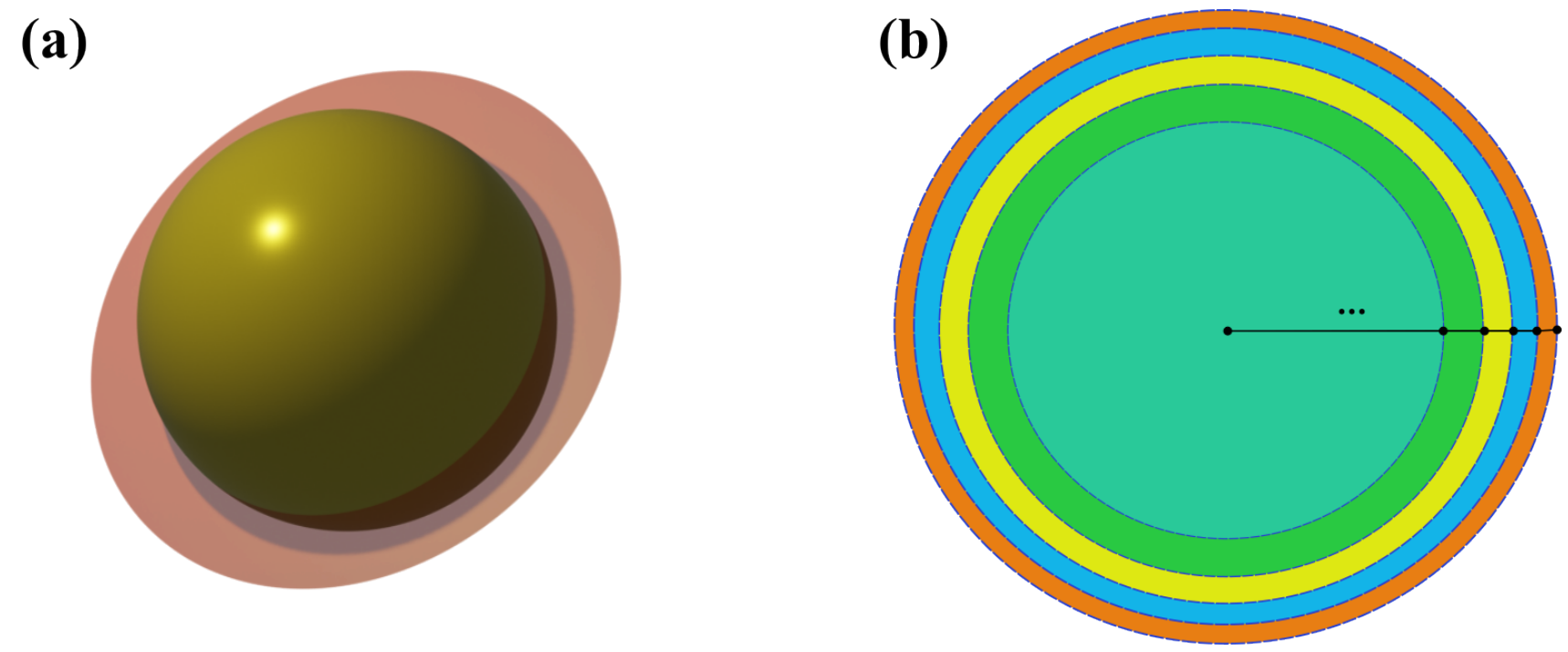

Figure S2: (a) The spherical nanoparticle of our problem. The shape of the geometrical elements are shown along a circular disk (a cut through the sphere). (b) The elements of the discretization along the slice shown in (a). The dots ... indicate an arbitrary number of elements between the origin and the first shell. The black linear segments, separated by black circles (vertices) are the geometric elements after analytical integration.

As nodes, we define the points of the discretization where a basis function takes the value 1, while all the rest vanish there. Then, the node supports this basis function (which in turn vanishes at the supporting nodes of the other basis functions in the element).

After performing integrations, the angular dependence is resolved in this manner, leading to an effective 1D discretization of the radial direction. Then (and from this point forward) the geometric elements are simply linear segments (see Figure S2(b)). Nodes may coincide with the geometric vertices (the endpoints) of an element. However this is not necessary. Elements and nodes pertaining to the source $\mathbf{P}(\mathbf{r})$ are denoted by (i, n) respectively with $(i, n) \in [1, 2, \ldots, N] \times [1, 2, \ldots, M]$, where N and M are natural numbers. On the other hand, testing elements and nodes are denoted by (j, ν) respectively with $(j, \nu) \in [1, 2, \ldots, N] \times [1, 2, \ldots, M]$.

We clarify that the indexing system presented here is a *local* one. This means that in each element we number the nodes as $1, 2, \ldots, M$ versus a *global* system where the nodes are counted from start to end across all elements. The indexing system per element will become clear by elaboration on the polynomials that resolve the radial dependence. These are [S11]

$$N_{i,n}(r) = \prod_{k=1,k\neq n}^{M} \frac{r - r_{i,k}}{r_{i,n} - r_{i,k}}. \tag{S23}$$

Once summed in each element, together with the appropriate coefficients which are calculated through the Method of Moments (MoM), they give the Lagrange interpolation polynomial of the unknown function. The use of such basis functions is both pragmatic (for a proof-of-concept we present here) and reasonable: The polarization density in each element should be analytical. In Figure S3, we visualize the polynomial basis functions and the supporting nodes in a single element.

All calculations (that is, the construction of *matrix* elements) are done in this system. As such, it would be convenient to express $\mathbf{P}(\mathbf{r})$ as

$$\mathbf{P}(\mathbf{r}) = \sum_{i=1}^{N}\sum_{n=1}^{M}\sum_{l=1}^{\infty}\sum_{m=-l}^{l} I^{(1)}_{i,n|l,m}\mathbf{g}^{(1)}_{i,n|l,m}(\mathbf{r}) + I^{(2)}_{i,n|l,m}\mathbf{g}^{(2)}_{i,n|l,m}(\mathbf{r}) + I^{(3)}_{i,n|l,m}\mathbf{g}^{(3)}_{i,n|l,m}(\mathbf{r}), \tag{S24}$$

where all the symbols have either been defined previously or in the main text. For later, we rewrite the basis and testing functions in this new indexing system

$$\begin{cases} \mathbf{g}^{(1)}_{i,n|l,m}(\mathbf{r}) = N_{i,n}(r)\mathbf{Y}_{lm}(\theta,\phi), & \mathbf{f}^{(1)}_{j,\nu|l',m'}(\mathbf{r}) = N_{j,\nu}(r)\mathbf{Y}_{l'm'}(\theta,\phi), \\ \mathbf{g}^{(2)}_{i,n|l,m}(\mathbf{r}) = N_{i,n}(r)\mathbf{Z}_{lm}(\theta,\phi), & \mathbf{f}^{(2)}_{j,\nu|l',m'}(\mathbf{r}) = N_{j,\nu}(r)\mathbf{Z}_{l'm'}(\theta,\phi), \\ \mathbf{g}^{(3)}_{i,n|l,m}(\mathbf{r}) = N_{i,n}(r)\mathbf{X}_{lm}(\theta,\phi), & \mathbf{f}^{(3)}_{j,\nu|l',m'}(\mathbf{r}) = N_{j,\nu}(r)\mathbf{X}_{l'm'}(\theta,\phi). \end{cases} \tag{S25}$$

We must note that there is overlap between common nodes of adjacent elements, e.g., for cubic polynomials $I^{(1)}_{i,4|l,m} = I^{(1)}_{i+1,1|l,m}$. We must map our local indexing system to a *global* one, which lists only the nodes of

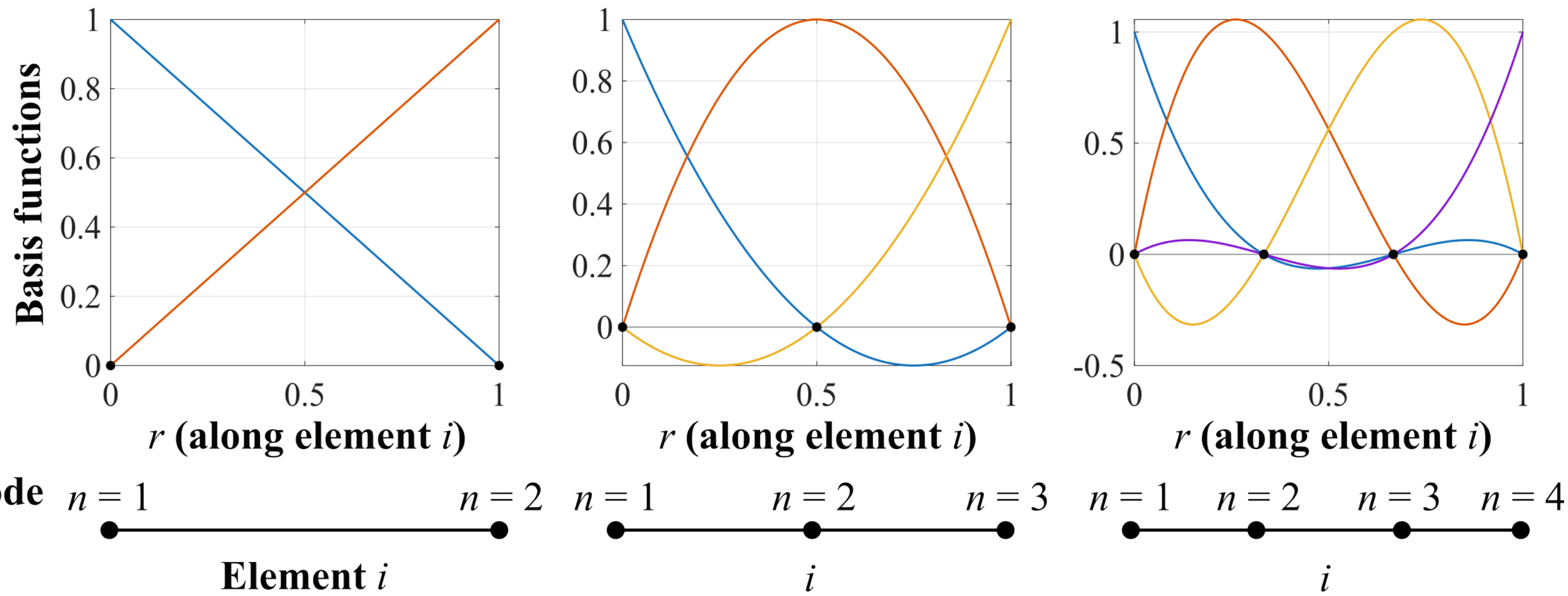

Figure S3: Basis functions for the radial resolution. (a) Linear, (b) quadratic, and (c) cubic basis functions have been implemented. The element on which the basis functions are defined is shown below the graphs. Depending on the order of the polynomials of the basis functions, nodes additional to the geometric ones (black circles) may be introduced.

discretization where the polarization density must be determined. These we term *computational nodes*. Their number is L and it ensues from the number of elements. In Table S1, we collect information on nodes, computational nodes, elements, and geometric vertices based on the order of basis functions.

Table S1: Explanation of the various systems of discretization in our method depending on the order of polynomials of the basis functions. The radial direction is discretized in N elements (linear segments) resulting in $N+1$ vertices. Depending on the order of the polynomials, different number of nodes exist in each element. This number affects directly the points on which the discretized $\mathbf{P}(\mathbf{r})$ is expected to be reconstructed. We name these points computational nodes.

Polynomials	# Elements	# Nodes per element	# Geometric vertices	# Computational nodes
Linear	N	$M = 2$	$N+1$	$N+1$
Quadratic	N	$M = 3$	$N+1$	$2N+1$
Cubic	N	$M = 4$	$N+1$	$3N+1$

The technical procedure to map from one system to the other is the *assembly*; here we follow the method delineated in [S10]. Through the assembly we recover from Equation (S24) the representation we use in the main text. We reiterate that we construct the separate matrix elements in the local system (see the next Section), assemble them, and invert the linear system to determine the coefficients I.

S7 Matrix elements

In this Section we construct a small dictionary for the matrix elements we use in our method, after the introduction of the discretization discussed in the previous Section. These are in full correspondence with Equations (S20) and (S22) of Section S5. We will show detailed derivations only for the right-hand side part of Equation (S20), the one that corresponds to the excitation. For the rest, the structure and the particular expressions will just be given; at the heart and justification of all lie the orthogonality properties of the scalar and vector spherical harmonics [S9], [S12], [S13].

Excitation term

We introduce the excitation vector $\mathbf{b}$ (for the symbols see also the main text) as a $3(M \cdot N) \times 1$ vector

$$\mathbf{b} = \begin{pmatrix} \mathbf{b}^{(1)} \\ \mathbf{b}^{(2)} \\ \mathbf{b}^{(3)} \end{pmatrix}, \ \mathbf{b}^{(1)} = \begin{pmatrix} b^{(1)}_{1,1} \\ b^{(1)}_{2,1} \\ \vdots \\ b^{(1)}_{N,1} \\ b^{(1)}_{1,2} \\ \vdots \\ b^{(1)}_{N,2} \\ \vdots \\ b^{(1)}_{j,\nu} \\ \vdots \\ b^{(1)}_{N,M} \end{pmatrix}, \ \mathbf{b}^{(2)} = \begin{pmatrix} b^{(2)}_{1,1} \\ b^{(2)}_{2,1} \\ \vdots \\ b^{(2)}_{N,1} \\ b^{(2)}_{1,2} \\ \vdots \\ b^{(2)}_{N,2} \\ \vdots \\ b^{(2)}_{j,\nu} \\ \vdots \\ b^{(2)}_{N,M} \end{pmatrix}, \ \mathbf{b}^{(3)} = \begin{pmatrix} b^{(3)}_{1,1} \\ b^{(3)}_{2,1} \\ \vdots \\ b^{(3)}_{N,1} \\ b^{(3)}_{1,2} \\ \vdots \\ b^{(3)}_{N,2} \\ \vdots \\ b^{(3)}_{j,\nu} \\ \vdots \\ b^{(3)}_{N,M} \end{pmatrix}. \tag{S26}$$

The excitation vector is a block vector, containing three $(M \cdot N) \times 1$ vectors $\mathbf{b}^{(1)}$, $\mathbf{b}^{(2)}$, $\mathbf{b}^{(3)}$ corresponding to the triad of testing functions, see Equation (S25). Each vector is organized as follows: for the νth (local) node (alternatively, basis function), all elements of the system are swept, up until we iterate through all ν nodes (for the rationale behind the data structure and for a visual representation, see Section S9). Let us focus on the elements of such vectors, namely $b^{(1,2,3)}_{j,\nu}$ (with $(1,2,3)$ as a superscript we denote thriftily quantities corresponding to the first, second, and third basis/testing functions). These are given by

$$b^{(1,2,3)}_{j,\nu} = \int_{V_j} \mathbf{f}^{(1,2,3)*}_{j,\nu|l',m'}(\mathbf{r}) \cdot \mathbf{E}_{\text{inc}}(\mathbf{r}) d\mathbf{r}, \tag{S27}$$

where V_j is the volume of the jth observation element (see Figure S2(b)). For the first testing function we have

$$b^{(1)}_{j,\nu} = \int_{V_j} N_{j,\nu}(r) \mathbf{Y}^*_{l'm'}(\theta,\phi) \cdot \mathbf{E}_{\text{inc}}(\mathbf{r}) d\mathbf{r}, \tag{S28}$$

substitute the Mie expression for the plane wave, Equation (S11), and expand the vector wave functions [S7] to get

$$\begin{aligned} b^{(1)}_{j,\nu} = & \int_{\Delta R_j} \int_0^{\pi} \int_0^{2\pi} r^2 \sin\theta d\phi d\theta dr N_{j,\nu}(r) \mathbf{Y}^*_{l'm'}(\theta,\phi) \cdot \\ & \sum_{l=1}^{\infty} \sum_{m=-l}^{l} a^{(\text{inc})}_{lm} j_l(k_0 r) \mathbf{X}_{lm}(\theta,\phi) + b^{(\text{inc})}_{lm} l(l+1) \frac{j_l(k_0 r)}{k_0 r} \mathbf{Y}_{lm}(\theta,\phi) + b^{(\text{inc})}_{lm} \frac{1}{k_0 r} \frac{\partial \left[r j_l(k_0 r) \right]}{\partial r} \mathbf{Z}_{lm}(\theta,\phi), \end{aligned} \tag{S29}$$

where ΔR_j is the length of the jth element and finally use orthogonality properties of vector spherical harmonics to analytically integrate along (θ,ϕ) and eventually achieve what we argued previously: simplification from a 3D to a 1D problem

$$b^{(1)}_{j,\nu} = l(l+1) b^{(\text{inc})}_{lm} \int_{\Delta R_j} \frac{j_l(k_0 r)}{k_0 r} N_{j,\nu}(r) r^2 dr. \tag{S30}$$

We carry out this (and all) integration numerically, as discussed in Section S10[6].

We proceed with the second testing function, the relevant terms of which are

$$b^{(2)}_{j,\nu} = \int_{V_j} N_{j,\nu}(r) \mathbf{Z}^*_{l'm'}(\theta,\phi) \cdot \mathbf{E}_{\text{inc}}(\mathbf{r}) d\mathbf{r}, \tag{S31}$$

[6] For implementation purposes, note that the simplification $r^2/r = r$ is performed; in this text we avoid it only to allow for straightforward separation of each term.

and can be simplified by the same tokens to[7]

$$b_{j,\nu}^{(2)} = l(l+1)b_{lm}^{(\mathrm{inc})} \int_{\Delta R_j} \frac{1}{k_0 r} \frac{\partial \left[r j_l(k_0 r) \right]}{\partial r} N_{j,\nu}(r) r^2 dr. \tag{S32}$$

Finally, for the third testing function

$$b_{j,\nu}^{(3)} = \int_{V_j} N_{j,\nu}(r) \mathbf{X}_{l'm'}^{*}(\theta,\phi) \cdot \mathbf{E}_{\mathrm{inc}}(\mathbf{r}) d\mathbf{r}, \tag{S33}$$

and

$$b_{j,\nu}^{(3)} = l(l+1)a_{lm}^{(\mathrm{inc})} \int_{\Delta R_j} j_l(k_0 r) N_{j,\nu}(r) r^2 dr. \tag{S34}$$

Naturally, the expressions given above are in the local indexing system. We perform assembly, mapping the $(M \cdot N) \times 1$ vectors to $L \times 1$ by means of a simple matrix multiplication, as follows

$$\mathbf{b}_{3L\times 1} = \begin{pmatrix} \mathbf{T}_{L\times(M\cdot N)} \mathbf{b}_{(M\cdot N)\times 1}^{(1)} \\ \mathbf{T}_{L\times(M\cdot N)} \mathbf{b}_{(M\cdot N)\times 1}^{(2)} \\ \mathbf{T}_{L\times(M\cdot N)} \mathbf{b}_{(M\cdot N)\times 1}^{(3)} \end{pmatrix}, \tag{S35}$$

where $\mathbf{T}$ (of dimensions shown in its subscript) is the transformation matrix.

Scattering term

The scattering term pertains to the fifth term of Equation (S20). It is arguably the most complicated term of our algorithm, consisting of double integrals (see Section S10), by virtue of the Green's function-meditated coupling between source and observation elements, and having frequency dependence. At the same time it is present irrespective of the level of sophistication of the material response, from LRA to SC-HDM (and even more advanced) simulations.

The scattering term is organized in a $3(M \cdot N) \times 3(M \cdot N)$ matrix as follows

$$\mathbf{S} = \left(\begin{array}{cc|c} \mathbf{S}^{(11)} & \mathbf{S}^{(12)} & \mathbf{0} \\ \mathbf{S}^{(21)} & \mathbf{S}^{(22)} & \mathbf{0} \\ \hline \mathbf{0} & \mathbf{0} & \mathbf{S}^{(33)} \end{array} \right), \tag{S36}$$

where $\mathbf{S}^{(11)}$, $\mathbf{S}^{(12)}$, $\mathbf{S}^{(21)}$, $\mathbf{S}^{(22)}$, $\mathbf{S}^{(33)}$, and $\mathbf{0}$ are $(M \cdot N) \times (M \cdot N)$ matrices, and multiplies from the left the coefficient vector (that is, the vector the coefficients $I_{i,n|l,m}^{(1,2,3)}$ are organized in)

$$\mathbf{I} = \left(\begin{array}{c} \mathbf{I}^{(1)} \\ \mathbf{I}^{(2)} \\ \hline \mathbf{I}^{(3)} \end{array} \right), \tag{S37}$$

where each of the vectors $\mathbf{I}^{(1)}$, $\mathbf{I}^{(2)}$, and $\mathbf{I}^{(3)}$ follows the same structuring as $\mathbf{b}^{(1)}$, $\mathbf{b}^{(2)}$, and $\mathbf{b}^{(3)}$. The lines in (S36) and (S37) separate clearly the coefficients corresponding to $\mathbf{Y}_{lm}$ and $\mathbf{Z}_{lm}$ from those of $\mathbf{X}_{lm}$. The structure above can be rigorously derived by the orthogonality arguments we mentioned earlier.

The organization of such matrices follows the same principle as that of $\mathbf{b}$ (from sets of testing/basis functions

[7] The derivatives of spherical Bessel functions are calculated by means of recurrence relations [S14].

to nodes and then to elements). The matrix elements of $\mathbf{S}^{(11)}$, $\mathbf{S}^{(12)}$, $\mathbf{S}^{(21)}$, $\mathbf{S}^{(22)}$, $\mathbf{S}^{(33)}$ are

$$\begin{cases} s^{(11)}_{ij,n\nu} = \tau \int_{\Delta R_j} \int_{\Delta R_i} N_{j,\nu}(r) N_{i,n}(r') \frac{\zeta_l(k_0 r)}{k_0 r} \frac{z_l(k_0 r')}{k_0 r'} r^2 (r')^2 dr' dr + \\ \qquad \frac{1}{\varepsilon_0} \int_{\Delta R_j} N_{j,\nu}(r) N_{i,n}(r) r^2 dr, \\ s^{(12)}_{ij,n\nu} = \tau \int_{\Delta R_j} \int_{\Delta R_i} N_{j,\nu}(r) N_{i,n}(r') \frac{\zeta_l(k_0 r)}{k_0 r} \frac{1}{k_0 r'} \frac{\partial [r' z_l(k_0 r')]}{\partial r'} r^2 (r')^2 dr' dr, \\ s^{(21)}_{ij,n\nu} = \tau \int_{\Delta R_j} \int_{\Delta R_i} N_{j,\nu}(r) N_{i,n}(r') \frac{1}{k_0 r} \frac{\partial [r \zeta_l(k_0 r)]}{\partial r} \frac{z_l(k_0 r')}{k_0 r'} r^2 (r')^2 dr' dr, \\ s^{(22)}_{ij,n\nu} = \tau \int_{\Delta R_j} \int_{\Delta R_i} N_{j,\nu}(r) N_{i,n}(r') \frac{1}{k_0 r} \frac{\partial [r \zeta_l(k_0 r)]}{\partial r} \frac{1}{k_0 r'} \frac{\partial [r' z_l(k_0 r')]}{\partial r'} r^2 (r')^2 dr' dr, \\ s^{(33)}_{ij,n\nu} = \tau \int_{\Delta R_j} \int_{\Delta R_i} N_{j,\nu}(r) N_{i,n}(r') \zeta_l(k_0 r) z_l(k_0 r') r^2 (r')^2 dr' dr, \end{cases} \tag{S38}$$

where $\tau = -i\omega^2 k_0 \mu_0 l(l+1)$. In the above, ζ and z can denote either spherical Bessel or Hankel functions (the particular conditions are described in [S9]) for the observation and source points respectively.

Finally, $\mathbf{S}$ must be appropriately mapped from the local indexing system to the global one of the L computational nodes. As before we perform assembly, which is slightly more complicated

$$\begin{cases} \mathbf{S}^{(11)}_{L\times L} = \mathbf{T}_{L\times(M\cdot N)} \mathbf{S}^{(11)}_{(M\cdot N)\times(M\cdot N)} \mathbf{T}^t_{L\times(M\cdot N)}, \\ \mathbf{S}^{(12)}_{L\times L} = \mathbf{T}_{L\times(M\cdot N)} \mathbf{S}^{(12)}_{(M\cdot N)\times(M\cdot N)} \mathbf{T}^t_{L\times(M\cdot N)}, \\ \mathbf{S}^{(21)}_{L\times L} = \mathbf{T}_{L\times(M\cdot N)} \mathbf{S}^{(21)}_{(M\cdot N)\times(M\cdot N)} \mathbf{T}^t_{L\times(M\cdot N)}, \\ \mathbf{S}^{(22)}_{L\times L} = \mathbf{T}_{L\times(M\cdot N)} \mathbf{S}^{(22)}_{(M\cdot N)\times(M\cdot N)} \mathbf{T}^t_{L\times(M\cdot N)}, \\ \mathbf{S}^{(33)}_{L\times L} = \mathbf{T}_{L\times(M\cdot N)} \mathbf{S}^{(33)}_{(M\cdot N)\times(M\cdot N)} \mathbf{T}^t_{L\times(M\cdot N)}, \end{cases} \tag{S39}$$

where t denotes the transpose of a matrix. The assembly is a universal procedure, that is, it applies to all matrices and vectors of our numerical recipe. For matrices of dimensions $(M \cdot N) \times (M \cdot N)$ the procedure is identical to (S39) and as such we suppress presenting it in the following, while stressing that it is applied to each term.

Local term

We evaluate next the "local" term, which is the fourth term of the left-hand side of Equation (S20). We call it local, since it is the only material term that appears in the LRA, the rest vanishing when $G[n] = 0$. This is the last term that appears in LRA (together with the scattering and the excitation one) and further the last that is frequency-dependent. It is quite interesting that classical terms appear more challenging numerically than nonclassical ones, at least in this respect and iteration of SC-HDM.

The contributions from the local term are organized in a $3(M \cdot N) \times 3(M \cdot N)$ matrix as follows

$$\mathbf{ML} = \left(\begin{array}{cc|c} \mathbf{ML}^{(11)} & \mathbf{0} & \mathbf{0} \\ \mathbf{0} & \mathbf{ML}^{(22)} & \mathbf{0} \\ \hline \mathbf{0} & \mathbf{0} & \mathbf{ML}^{(33)} \end{array} \right), \tag{S40}$$

where $\mathbf{ML}^{(11)}$, $\mathbf{ML}^{(22)}$, and $\mathbf{ML}^{(33)}$ are $(M \cdot N) \times (M \cdot N)$ matrices with elements given by

$$\begin{cases} ml^{(11)}_{ij,n\nu} = \frac{1}{\varepsilon_0} \int_{\Delta R_j} \frac{1}{\chi(r,\omega)} N_{j,\nu}(r) N_{i,n}(r) r^2 dr, \\ ml^{(22)}_{ij,n\nu} = \frac{l(l+1)}{\varepsilon_0} \int_{\Delta R_j} \frac{1}{\chi(r,\omega)} N_{j,\nu}(r) N_{i,n}(r) r^2 dr, \\ ml^{(33)}_{ij,n\nu} = \frac{l(l+1)}{\varepsilon_0} \int_{\Delta R_j} \frac{1}{\chi(r,\omega)} N_{j,\nu}(r) N_{i,n}(r) r^2 dr. \end{cases} \tag{S41}$$

$\mathbf{ML}$ formally acts on $\mathbf{I}$ and it is clear that there is no coupling between the coefficients in this case.

Thomas-Fermi term

We turn our attention to the Thomas-Fermi term of the first integral of the left-hand side of Equation (S20). Such contributions are organized in a $2(M \cdot N) \times 2(M \cdot N)$ matrix

$$\mathbf{TF} = \begin{pmatrix} \mathbf{TF}^{(11)} & \mathbf{TF}^{(12)} \\ \mathbf{TF}^{(21)} & \mathbf{TF}^{(22)} \end{pmatrix}, \tag{S42}$$

where $\mathbf{TF}^{(11)}$, $\mathbf{TF}^{(12)}$, $\mathbf{TF}^{(21)}$, and $\mathbf{TF}^{(22)}$ are $(M \cdot N) \times (M \cdot N)$ matrices. We underline that such terms include the divergence of the testing functions, but also the divergence of the basis functions. It can be proven that both vanish for the third testing/basis functions, which thus do not enter into discussion (of course it is possible for $\mathbf{TF}$ to act on the whole $\mathbf{I}$ by zero filling). This applies to all terms that include the divergence of basis functions (see below). The matrix elements of each of the submatrices are

$$\begin{cases} tf^{(11)}_{ij,n\nu} = \int_{\Delta R_j} \gamma_{\mathrm{TF}}(r) \left(\frac{2}{r}N_{j,\nu}(r) + \frac{dN_{j,\nu}(r)}{dr}\right)\left(\frac{2}{r}N_{i,n}(r) + \frac{dN_{i,n}(r)}{dr}\right) r^2 dr, \\ tf^{(12)}_{ij,n\nu} = -\int_{\Delta R_j} \gamma_{\mathrm{TF}}(r) \left(\frac{2}{r}N_{j,\nu}(r) + \frac{dN_{j,\nu}(r)}{dr}\right) \frac{l(l+1)}{r} N_{i,n}(r) r^2 dr, \\ tf^{(21)}_{ij,n\nu} = -\int_{\Delta R_j} \gamma_{\mathrm{TF}}(r) \frac{l(l+1)}{r} N_{j,\nu}(r) \left(\frac{2}{r}N_{i,n}(r) + \frac{dN_{i,n}(r)}{dr}\right) r^2 dr, \\ tf^{(22)}_{ij,n\nu} = \int_{\Delta R_j} \gamma_{\mathrm{TF}}(r) \frac{l^2(l+1)^2}{r^2} N_{j,\nu}(r) N_{i,n}(r) r^2 dr. \end{cases} \tag{S43}$$

Above, we have absorbed the constant $1/e^2$ of the first term of Equation (S20) in γ_{TF}.

XC term

The next term originates from the XC contribution of G. It is the second term of the first term of the left-hand side of Equation (S20). The XC contributions are organized in a $2(M \cdot N) \times 2(M \cdot N)$ matrix

$$\mathbf{XC} = \begin{pmatrix} \mathbf{XC}^{(11)} & \mathbf{XC}^{(12)} \\ \mathbf{XC}^{(21)} & \mathbf{XC}^{(22)} \end{pmatrix}, \tag{S44}$$

where $\mathbf{XC}^{(11)}$, $\mathbf{XC}^{(12)}$, $\mathbf{XC}^{(21)}$, and $\mathbf{XC}^{(22)}$ are $(M \cdot N) \times (M \cdot N)$ matrices, whose elements are given by

$$\begin{cases} xc^{(11)}_{ij,n\nu} = \int_{\Delta R_j} \gamma_{\mathrm{XC}}(r) \left(\frac{2}{r}N_{j,\nu}(r) + \frac{dN_{j,\nu}(r)}{dr}\right)\left(\frac{2}{r}N_{i,n}(r) + \frac{dN_{i,n}(r)}{dr}\right) r^2 dr, \\ xc^{(12)}_{ij,n\nu} = -\int_{\Delta R_j} \gamma_{\mathrm{XC}}(r) \left(\frac{2}{r}N_{j,\nu}(r) + \frac{dN_{j,\nu}(r)}{dr}\right) \frac{l(l+1)}{r} N_{i,n}(r) r^2 dr, \\ xc^{(21)}_{ij,n\nu} = -\int_{\Delta R_j} \gamma_{\mathrm{XC}}(r) \frac{l(l+1)}{r} N_{j,\nu}(r) \left(\frac{2}{r}N_{i,n}(r) + \frac{dN_{i,n}(r)}{dr}\right) r^2 dr, \\ xc^{(22)}_{ij,n\nu} = \int_{\Delta R_j} \gamma_{\mathrm{XC}}(r) \frac{l^2(l+1)^2}{r^2} N_{j,\nu}(r) N_{i,n}(r) r^2 dr. \end{cases} \tag{S45}$$

Once more, the constant $1/e^2$ of the first term of Equation (S20) is absorbed in γ_{XC}.

First von Weizsäcker term

This term shares the same structure as the $\mathbf{TF}$ and $\mathbf{XC}$ terms. and originates from the von Weizsäcker contribution of G, the third term of the first term of the left-hand side of Equation (S20). It is organized as

$${}_1\mathbf{vW} = \begin{pmatrix} {}_1\mathbf{vW}^{(11)} & {}_1\mathbf{vW}^{(12)} \\ {}_1\mathbf{vW}^{(21)} & {}_1\mathbf{vW}^{(22)} \end{pmatrix}, \tag{S46}$$

where ${}_1\mathbf{vW}$ is a $2(M \cdot N) \times 2(M \cdot N)$ matrix and its submatrices ${}_1\mathbf{vW}^{(11)}$, ${}_1\mathbf{vW}^{(12)}$, ${}_1\mathbf{vW}^{(21)}$, and ${}_1\mathbf{vW}^{(22)}$ are each $(M \cdot N) \times (M \cdot N)$. Their matrix elements are

$$\begin{cases} {}_1vw^{(11)}_{ij,n\nu} = \displaystyle\int_{\Delta R_j} \gamma_{\mathrm{vW}}(r) \left(\frac{2}{r}N_{j,\nu}(r) + \frac{dN_{j,\nu}(r)}{dr}\right)\left(\frac{2}{r}N_{i,n}(r) + \frac{dN_{i,n}(r)}{dr}\right) r^2 dr, \\ {}_1vw^{(12)}_{ij,n\nu} = -\displaystyle\int_{\Delta R_j} \gamma_{\mathrm{vW}}(r) \left(\frac{2}{r}N_{j,\nu}(r) + \frac{dN_{j,\nu}(r)}{dr}\right)\frac{l(l+1)}{r} N_{i,n}(r) r^2 dr, \\ {}_1vw^{(21)}_{ij,n\nu} = -\displaystyle\int_{\Delta R_j} \gamma_{\mathrm{vW}}(r) \frac{l(l+1)}{r} N_{j,\nu}(r) \left(\frac{2}{r}N_{i,n}(r) + \frac{dN_{i,n}(r)}{dr}\right) r^2 dr, \\ {}_1vw^{(22)}_{ij,n\nu} = \displaystyle\int_{\Delta R_j} \gamma_{\mathrm{vW}}(r) \frac{l^2(l+1)^2}{r^2} N_{j,\nu}(r) N_{i,n}(r) r^2 dr, \end{cases} \tag{S47}$$

where the constant $1/e^2$ of the first term of Equation (S20) is absorbed in $\gamma_{\mathrm{vW},1}$.

Second von Weizsäcker term

It is clear that this term concerns the auxiliary function $\mathbf{F}(\mathbf{r})$ rather than $\mathbf{P}(\mathbf{r})$. In order to construct a matrix system, we expand $\mathbf{F}(\mathbf{r})$ in a series of basis functions and use the indexing system we have detailed before. To this end, we write

$$\mathbf{F}(\mathbf{r}) = \sum_{\rho=1}^{N}\sum_{\psi=1}^{M}\sum_{\xi=1}^{\infty}\sum_{\upsilon=-\xi}^{\xi} d^{(1)}_{\rho,\psi|\xi,\upsilon}\mathbf{w}^{(1)}_{\rho,\psi|\xi,\upsilon}(\mathbf{r}) + d^{(2)}_{\rho,\psi|\xi,\upsilon}\mathbf{w}^{(2)}_{\rho,\psi|\xi,\upsilon}(\mathbf{r}), \tag{S48}$$

where ρ plays the role of i, ψ of n, ξ of l, and υ of m. Further $d^{(1,2)}_{\rho,\psi|\xi,\upsilon}$ are the coefficients to be determined, and $\mathbf{w}^{(1,2)}_{\rho,\psi|\xi,\upsilon}$ are the basis functions, which are given by Equation (S25). Since Equation (S16) links $\mathbf{F}(\mathbf{r})$ with $\mathbf{P}(\mathbf{r})$, the indices of the expansions of the two functions cannot be independent; yet for generality we use different symbols. In Equation (S48), this link shows: since $\mathbf{F}$ is determined through a (gradient of) divergence of $\mathbf{P}(\mathbf{r})$ the third basis functions, which are divergence free, are not used.

The coefficients $d^{(1,2)}_{\rho,\psi|\xi,\upsilon}$ can be formally organized in a $2(M \cdot N) \times 1$ vector as follows

$$\mathbf{d} = \begin{pmatrix} \mathbf{d}^{(1)} \\ \mathbf{d}^{(2)} \end{pmatrix}. \tag{S49}$$

The second von Weizsäcker term (the second term of the left-hand side of Equation (S20)) acts on them; the contributions of this term are organized as

$${}_2\mathbf{vW} = \begin{pmatrix} {}_2\mathbf{vW}^{(11)} & \mathbf{0} \\ {}_2\mathbf{vW}^{(21)} & \mathbf{0} \end{pmatrix}, \tag{S50}$$

where ${}_2\mathbf{vW}$ is a $2(M \cdot N) \times 2(M \cdot N)$ matrix and ${}_2\mathbf{vW}^{(11)}$ and ${}_2\mathbf{vW}^{(21)}$ are $(M \cdot N) \times (M \cdot N)$ matrices, whose elements are

$$\begin{cases} {}_2vw^{(11)}_{ij,n\nu} = \displaystyle\int_{V_j} |\boldsymbol{\gamma}_{vW,2}(r)| \left(\frac{2}{r}N_{j,\nu}(r) + \frac{dN_{j,\nu}(r)}{dr}\right) N_{i,n}(r) r^2 dr, \\ {}_2vw^{(21)}_{ij,n\nu} = -\displaystyle\int_{V_j} |\boldsymbol{\gamma}_{vW,2}(r)| \frac{l(l+1)}{r} N_{j,\nu}(r) N_{i,n}(r) r^2 dr, \end{cases} \tag{S51}$$

where we resumed using the previous indices i, n, l, and m (as the determination of $\mathbf{F}(\mathbf{r})$ and $\mathbf{P}(\mathbf{r})$ must be consistent). As per usual, the constant $1/e^2$ of the second term of Equation (S20) is absorbed in $\boldsymbol{\gamma}_{\mathrm{vW},2}$.

Third von Weizsäcker term

This term concerns also $\mathbf{F}(\mathbf{r})$. It pertains to the third term of the left-hand side of Equation (S20). It is organized as

$${}_3\mathbf{vW} = \begin{pmatrix} {}_3\mathbf{vW}^{(11)} & {}_3\mathbf{vW}^{(12)} \\ {}_3\mathbf{vW}^{(21)} & {}_3\mathbf{vW}^{(22)} \end{pmatrix}, \tag{S52}$$

where ${}_3\mathbf{vW}$ is a $2(M\cdot N)\times 2(M\cdot N)$ matrix and ${}_3\mathbf{vW}^{(11)}$, ${}_3\mathbf{vW}^{(12)}$, ${}_3\mathbf{vW}^{(21)}$, and ${}_3\mathbf{vW}^{(21)}$ are $(M\cdot N)\times(M\cdot N)$ matrices with elements

$$\begin{cases} {}_3vw^{(11)}_{ij,n\nu} = \int_{\Delta R_j} \gamma_{\text{vW},3}(r)\left(\frac{2}{r}N_{j,\nu}(r)+\frac{dN_{j,\nu}(r)}{dr}\right)\left(\frac{2}{r}N_{i,n}(r)+\frac{dN_{i,n}(r)}{dr}\right)r^2dr, \\ {}_3vw^{(12)}_{ij,n\nu} = -\int_{\Delta R_j} \gamma_{\text{vW},3}(r)\left(\frac{2}{r}N_{j,\nu}(r)+\frac{dN_{j,\nu}(r)}{dr}\right)\frac{l(l+1)}{r}N_{i,n}(r)r^2dr, \\ {}_3vw^{(21)}_{ij,n\nu} = -\int_{\Delta R_j} \gamma_{\text{vW},3}(r)\frac{l(l+1)}{r}N_{j,\nu}(r)\left(\frac{2}{r}N_{i,n}(r)+\frac{dN_{i,n}(r)}{dr}\right)r^2dr, \\ {}_3vw^{(22)}_{ij,n\nu} = \int_{\Delta R_j} \gamma_{\text{vW},3}(r)\frac{l^2(l+1)^2}{r^2}N_{j,\nu}(r)N_{i,n}(r)r^2dr. \end{cases} \tag{S53}$$

The constant $1/e^2$ of the third term of Equation (S20) is absorbed in $\gamma_{\text{vW},3}$.

First auxiliary term

We proceed with the auxiliary Equation (S22) and its first term of the left-hand side. It is organized as a $2(M\cdot N)\times 2(M\cdot N)$ matrix

$$\mathbf{FF} = \begin{pmatrix} \mathbf{FF}^{(11)} & \mathbf{0} \\ \mathbf{0} & \mathbf{FF}^{(22)} \end{pmatrix}, \tag{S54}$$

which acts on $\mathbf{d}$ and where $\mathbf{FF}^{(11)}$ and $\mathbf{FF}^{(22)}$ are $(M\cdot N)\times(M\cdot N)$ matrices with elements

$$\begin{cases} ff^{(11)}_{ij,n\nu} = \int_{\Delta R_j} N_{j,\nu}(r)N_{i,n}(r)r^2dr, \\ ff^{(22)}_{ij,n\nu} = l(l+1)\int_{\Delta R_j} N_{j,\nu}(r)N_{i,n}(r)r^2dr. \end{cases} \tag{S55}$$

Second auxiliary term

The final term of our discussion pertains to the second term of the left-hand side of Equation (S22). It is organized as a $2(M\cdot N)\times 2(M\cdot N)$ matrix

$$\mathbf{DF} = \begin{pmatrix} \mathbf{DF}^{(11)} & \mathbf{DF}^{(12)} \\ \mathbf{DF}^{(21)} & \mathbf{DF}^{(22)} \end{pmatrix}, \tag{S56}$$

where $\mathbf{DF}^{(11)}$, $\mathbf{DF}^{(12)}$, $\mathbf{DF}^{(21)}$ and $\mathbf{DF}^{(22)}$ are $(M\cdot N)\times(M\cdot N)$ matrices with elements

$$\begin{cases} df^{(11)}_{ij,n\nu} = \int_{\Delta R_j} \left(\frac{2}{r}N_{j,\nu}(r)+\frac{dN_{j,\nu}(r)}{dr}\right)\left(\frac{2}{r}N_{i,n}(r)+\frac{dN_{i,n}(r)}{dr}\right)r^2dr, \\ df^{(12)}_{ij,n\nu} = -\int_{\Delta R_j} \left(\frac{2}{r}N_{j,\nu}(r)+\frac{dN_{j,\nu}(r)}{dr}\right)\frac{l(l+1)}{r}N_{i,n}(r)r^2dr, \\ df^{(21)}_{ij,n\nu} = -\int_{\Delta R_j} \frac{l(l+1)}{r}N_{j,\nu}(r)\left(\frac{2}{r}N_{i,n}(r)+\frac{dN_{i,n}(r)}{dr}\right)r^2dr, \\ df^{(22)}_{ij,n\nu} = \int_{\Delta R_j} \frac{l^2(l+1)^2}{r^2}N_{j,\nu}(r)N_{i,n}(r)r^2dr. \end{cases} \tag{S57}$$

$\mathbf{DF}$ applies on $\mathbf{I}$. We note that while formally we use different testing functions (i.e., different indices, but same functional form) the final results are given in the familiar indexing system. The individual calculations are independent from each other and as such there is no violation should *the final result* be presented in a single indexing system.

S8 Linear system

The effort above culminates with the presentation of a concise linear system, which is to be solved. Using our symbolism this is

$$\begin{cases}(\mathbf{TF}+\mathbf{XC}+{}_1\mathbf{vW}+\mathbf{ML}+\mathbf{S})\mathbf{I}+({}_2\mathbf{vW}+{}_3\mathbf{vW})\mathbf{d}=\mathbf{b},\\ \mathbf{FFd}+\mathbf{DFI}=\mathbf{0},\end{cases} \tag{S58}$$

and eliminating $\mathbf{d}$ above yields

$$\boxed{(\mathbf{TF}+\mathbf{XC}+{}_1\mathbf{vW}+\mathbf{ML}+\mathbf{S})\mathbf{I}-({}_2\mathbf{vW}+{}_3\mathbf{vW})\mathbf{FF}^{-1}\mathbf{DFI}=\mathbf{b}.} \tag{S59}$$

This system is inverted and yields the coefficient vector $\mathbf{I}$ from which the full response of the scatterer can be determined[8].

In terms of the implementation, and following from the previous Section, it is clear that Equation (S59) includes two systems independent of each other: one for $\mathbf{I}^{(3)}$ and one for $\mathbf{I}^{(1)}, \mathbf{I}^{(2)}$. These are

$$(\mathbf{ML}^{(33)}+\mathbf{S}^{(33)})\mathbf{I}^{(3)}=\mathbf{b}^{(3)}, \tag{S60}$$

which note that it is identical to a fully classical approach, and

$$\begin{pmatrix}\mathbf{TF}^{(11)}+\mathbf{XC}^{(11)}+{}_1\mathbf{vW}^{(11)}+\mathbf{ML}^{(11)}+\mathbf{S}^{(11)} & \mathbf{TF}^{(12)}+\mathbf{XC}^{(12)}+{}_1\mathbf{vW}^{(12)}+\mathbf{S}^{(12)}\\ \mathbf{TF}^{(21)}+\mathbf{XC}^{(21)}+{}_1\mathbf{vW}^{(21)}+\mathbf{S}^{(21)} & \mathbf{TF}^{(22)}+\mathbf{XC}^{(22)}+{}_1\mathbf{vW}^{(22)}+\mathbf{ML}^{(22)}+\mathbf{S}^{(22)}\end{pmatrix}\begin{pmatrix}\mathbf{I}^{(1)}\\ \mathbf{I}^{(2)}\end{pmatrix}$$
$$-\begin{pmatrix}{}_2\mathbf{vW}^{(11)}+{}_3\mathbf{vW}^{(11)} & {}_3\mathbf{vW}^{(12)}\\ {}_2\mathbf{vW}^{(21)}+{}_3\mathbf{vW}^{(21)} & {}_3\mathbf{vW}^{(22)}\end{pmatrix}\begin{pmatrix}\left[\mathbf{FF}^{(11)}\right]^{-1} & \mathbf{0}\\ \mathbf{0} & \left[\mathbf{FF}^{(22)}\right]^{-1}\end{pmatrix}\begin{pmatrix}\mathbf{DF}^{(11)} & \mathbf{DF}^{(12)}\\ \mathbf{DF}^{(21)} & \mathbf{DF}^{(22)}\end{pmatrix}\begin{pmatrix}\mathbf{I}^{(1)}\\ \mathbf{I}^{(2)}\end{pmatrix}=\begin{pmatrix}\mathbf{b}^{(1)}\\ \mathbf{b}^{(2)}\end{pmatrix}. \tag{S61}$$

Computationally, acknowledging such behavior allows to invert two systems, of dimensions $L \times L$ and $2L \times 2L$ respectively, in place of a single one of much larger dimensions, $3L \times 3L$, especially beneficial for more accurate (of larger L) simulations.

S9 Levels of organization

We find it particularly useful to return to our data format and discuss the various levels of organization (for a preliminary discussion, see Section S6). The choice is important for the performance of the method as well.

The various levels of organization, starting from the three sets of basis/testing functions, to the nodes of a single element, and then to all elements of the particular node (e.g., first, second) are shown in Figure S4.

In terms of the performance, the choice of sweeping first the nodes and then the elements (though the opposite is also possible) is particularly beneficial in our implementation: the latter sweep is performed through vectorization, while the first is implemented in a simple scalar manner (for loop); the number of nodes is very small and as such the sweep does not harm the performance (in contrast to sweeping in a scalar manner through the potentially hundreds of elements).

S10 Numerical integration

All integrations are performed numerically using a simple Q^{th} order Gauss-Legendre integration [S15]. This is a pragmatic choice, as it offers fast computation (in comparison to, for example, built-in MATLAB methods) and is well-suited to the analytic integrands we are handling. A reasonable objection can be raised for the case $r = 0$ and the spherical Hankel functions of Equation (S38). As long as the integration order Q is finite and not too large, the sampling points are sufficiently far from $r = 0$ (and as such no pathological behavior was observed).

[8] Notice that in the main text, Equation (13), we absorbed the term $\mathbf{FF}^{-1}\mathbf{DF}$ in $\mathbf{AUX}$ for a clearer presentation.

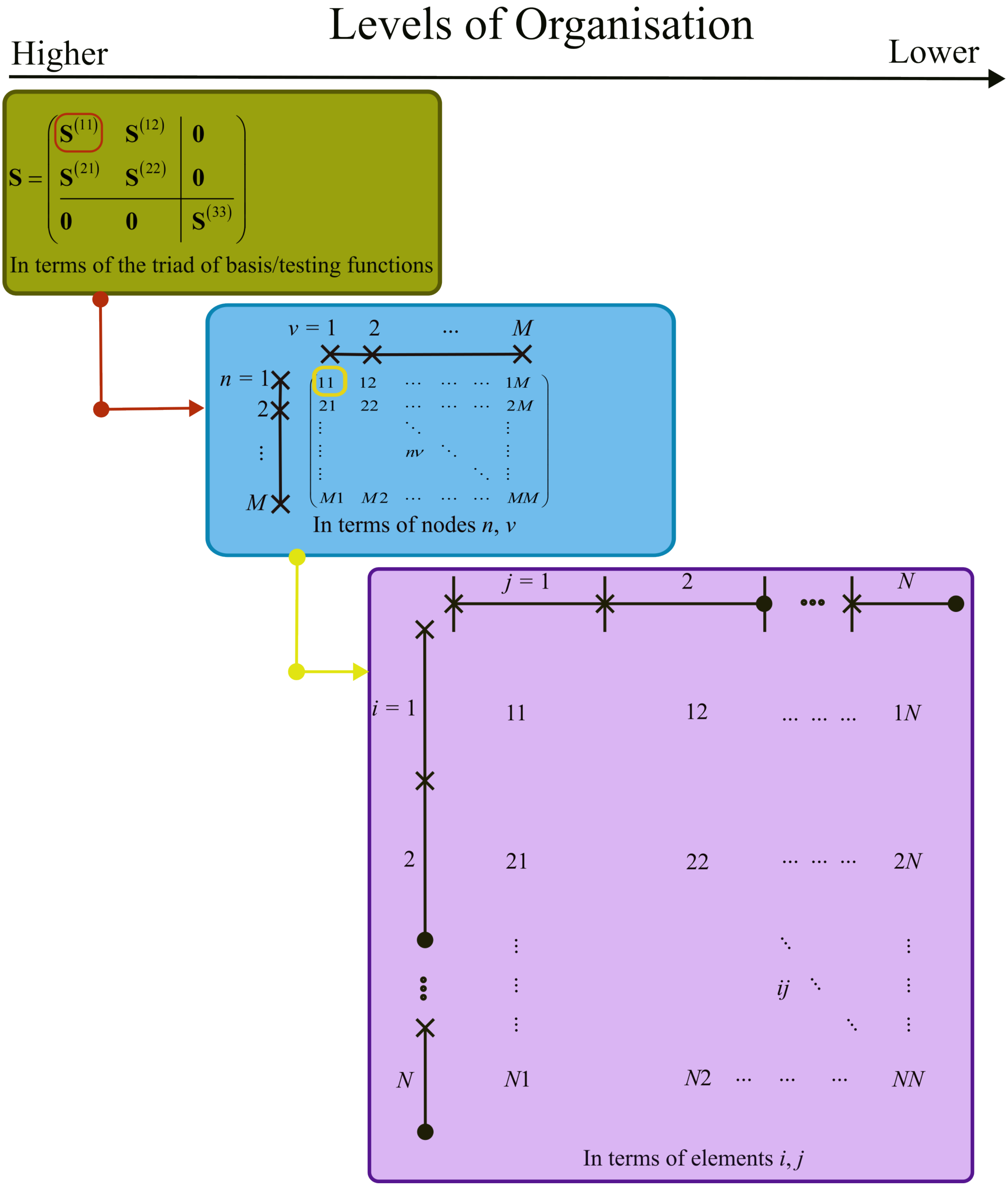

Figure S4: Organization of all matrices. The first level of organization pertains (olive green frame) to the three distinct sets of basis/testing functions. Each submatrix is an $M \times M$ matrix organized in terms of the nodes ("x") of a single element (black lines). This is the second level (blue frame). Finally, each matrix element consists of an $N \times N$ matrix, which contains information across all elements for the basis functions indicated by the second level. This is the third and final level (purple frame).

Further, the use of a consistent rule throughout (and if possible) results to a uniform (and simple) programming. We note that certain integrals, such as those of the excitation terms (i.e., Equations (S30), (S32), and (S34)), can be analytically determined. Still, we opt for a numerical approach. For one, for the reasons already listed, and for another, because the result involves generalized hypergeometric functions. The implementation thereof exists in MATLAB, however the performance is inferior in this case.

We would like further to elaborate on the way we conduct our integrations. For the material and excitation contributions, the single integral is conducted with respect only to observation elements. Due to the subdomain character of the basis functions, all such material terms result in diagonal matrices (while the excitation is gathered in a vector) in the element level (see the purple frame of Figure S4).

The only full matrix is the one pertaining to the scattering contribution (notice that it is also symmetric). We conduct the integration differently depending on the position of the element on the matrix. In more detail, for $|\mathbf{r}'| > |\mathbf{r}|$ (which pertains to elements in the first subdiagonal and below), and using $s^{(33)}_{ij,n\nu}$ for demonstration,

$$s^{(33)}_{ij,n\nu} = \tau \int_{\Delta R_j} \int_{\Delta R_i} N_{j,\nu}(r) N_{i,n}(r') j_l(k_0 r) h_l^{(1)}(k_0 r') r^2 (r')^2 dr' dr, \tag{S62}$$

or

$$s^{(33)}_{ij,n\nu} = \tau \int_{\Delta R_j} N_{j,\nu}(r) j_l(k_0 r) r^2 dr \int_{\Delta R_i} N_{i,n}(r') h_l^{(1)}(k_0 r')(r')^2 dr', \tag{S63}$$

since there is no overlap between the integration regions. Instead of a double numerical integration, we calculate the product of two single numerical integrations. The same applies if $|\mathbf{r}| > |\mathbf{r}'|$. The situation is more complicated (as per usual in a MoM [S16]), when $|\mathbf{r}| = |\mathbf{r}'|$, that is, for the diagonal elements of the matrix (also known as self terms). We study $s^{(33)}_{ij,n\nu}$ on a single element $\Delta R_i = \Delta R_j = r_2^i - r_1^i$. This is

$$\begin{aligned} s^{(33)}_{ij,n\nu} =& \tau \int_{r_1^i}^{r_2^i} N_{i,\nu}(r) h_l^{(1)}(k_0 r) r^2 \int_{r_1^i}^{r} N_{i,n}(r') j_l(k_0 r')(r')^2 dr' dr + \\ & \tau \int_{r_1^i}^{r_2^i} N_{i,\nu}(r) j_l(k_0 r) r^2 \int_{r}^{r_2^i} N_{i,n}(r') h_l^{(1)}(k_0 r')(r')^2 dr' dr. \end{aligned} \tag{S64}$$

We interchange the order of integration in the first double integral and change variables (setting $r' \longleftrightarrow r$), resulting in

$$\begin{aligned} s^{(33)}_{ij,n\nu} =& \tau \int_{r_1^i}^{r_2^i} N_{i,n}(r) j_l(k_0 r) r^2 \int_{r}^{r_2^i} N_{i,\nu}(r') h_l^{(1)}(k_0 r')(r')^2 dr' dr + \\ & \tau \int_{r_1^i}^{r_2^i} N_{i,\nu}(r) j_l(k_0 r) r^2 \int_{r}^{r_2^i} N_{i,n}(r') h_l^{(1)}(k_0 r')(r')^2 dr' dr. \end{aligned} \tag{S65}$$

This is the sole double numerical integration of our solution strategy.

S11 On the application of the boundary conditions

SC-HDM has an interesting behavior: beyond the so-called critical frequency, it predicts induced electron densities that do not decay (exponentially) with distance, but rather as damped oscillations [S17]. This results in a particularly verbose optical spectrum (beyond the aforementioned frequency), where, as the spill-out allowance s is increased (the space increases) more and more (unphysical) Bennett resonances appear. Though as $s \to \infty$ physical behavior is recovered, this is a clearly undesirable characteristic of the model. These findings, initially reported by a Finite Element Method [S2], [S17], have been reproduced by our Volume Integral Equation (VIE) approach (see Figure S5), cementing that it is a characteristic of SC-HDM regardless of numerical treatment. Note that from the perspective of classical Computational Electromagnetics (CEM), this is a peculiar finding: VIE methods have a well defined computational domain, that of the scatterer. However, *the scatterer* in SC-HDM is nominally

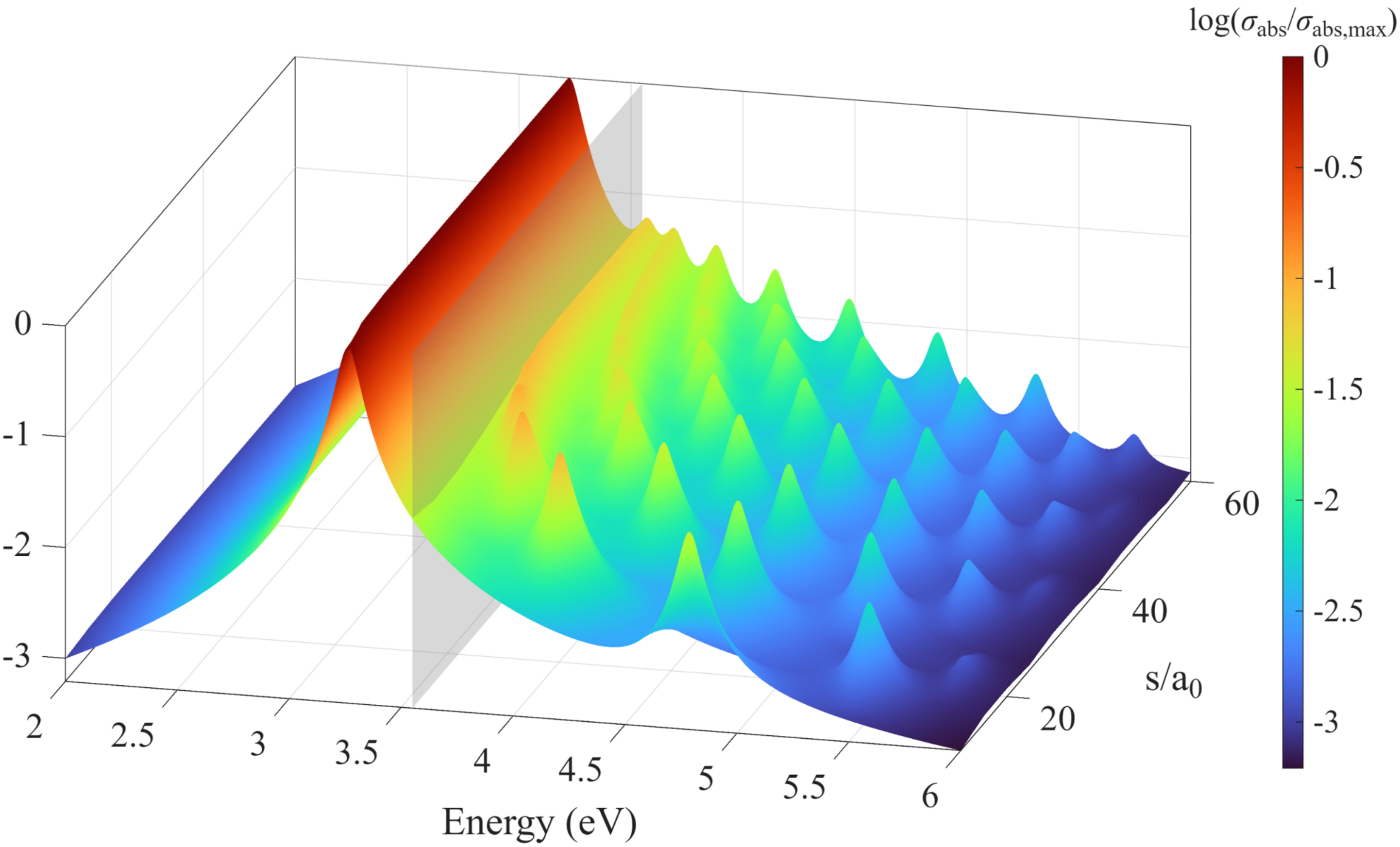

Figure S5: Normalized absorption cross section with increasing spill-out allowance s (multiple of the Bohr radius a_0). The spectrum becomes particularly interesting beyond the critical frequency (here indicated with a gray plane and its value taken from [S17]): as s increases, Bennett resonances multiply. For large s a shoulder starts to be formed. Clearly, the calculation is not well-converged. The opposite behavior is observed below the critical frequency, with the well-formed dipole resonance: domain effects are negligible there.

infinite, a radical departure from the classical perspective, which translates to this unusual behavior that clearly distinguishes CEM from its nascent Mesoscopic subbranch.

It is clear from the discussion above that it is hard to formulate accurate boundary conditions. Further, the boundary conditions influence appreciably the optical spectra [S18]. For example, the approach of [S2], [S17] where $\mathbf{P} = \mathbf{0}$ at the interface ($r = R + s$) is applied, is well-motivated below the plasma frequency (where exponentially decaying charge densities are induced), but not beyond it (due to the aforementioned oscillatory behavior). In our approach, the same boundary condition is applied *implicitly*. The boundary terms of Equation (S18) and (S21), occurring from integration by parts, are

$$-\frac{1}{e}\oint_S \hat{\mathbf{n}}\cdot\tilde{\mathbf{P}}(\mathbf{r})\left(\frac{\delta G[n]}{\delta n}\right)_1 dS, \quad -\oint_S \hat{\mathbf{n}}\cdot\tilde{\mathbf{F}}(\mathbf{r})\nabla\cdot\mathbf{P}(\mathbf{r})dS,$$

where $S = S_{\text{in}} \cup S_{\text{out}}$ is the surface of each element (here referring to the spherical shells, see Figure S2) including its inner and outer interface and $\hat{\mathbf{n}}$ is the unit normal directed outwards. Our line of reasoning on why the two integrals above vanish, differs based on the element considered. From Figure S6, it is clear that the contribution from the outer surface of element $j-1$ is canceled from the one of the inner surface of element j. This is tantamount to accepting continuity of testing/basis functions across elements. The only exception is the contribution of the outer surface of the last element; this one cannot be just canceled. For the SC-HDM, we assume that the polarization density $\mathbf{P}$ (and its derivatives) vanish there by virtue of being sufficiently far from the jellium edge. Note that we do not enforce this, but anticipate it from the dynamics of the system (as such, we remove the surface terms from the formalism, but do not set numerically $\mathbf{P} = \mathbf{0}$). For the HDM, where the second surface integral is not necessary and the first involves only n_1, we invoke the Sauter Additional Boundary Condition (ABC) [S19] and enforce the elimination of the integral on the interface $r = R$. Finally, the above discussion is not meaningful for the LRA, since no surface integrals are involved there. We return to the SC-HDM,

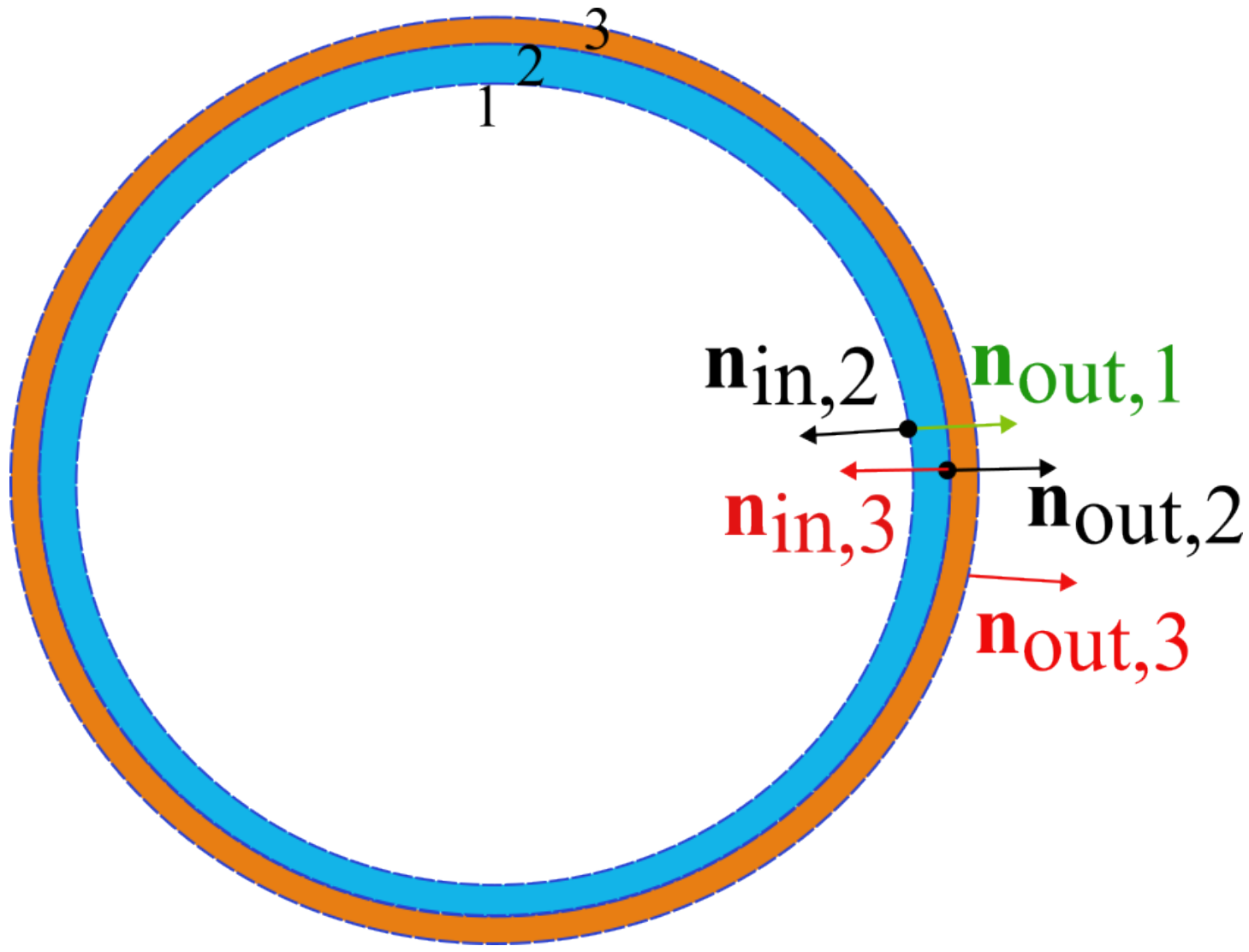

Figure S6: Cancellation of surface contributions in successive elements. Three elements $(1, 2, 3)$ with their surface normals (from in to out) attached to their inner and outer surfaces are shown. It is clear that, on each surface, the contributions are equal and opposite, leading to cancellation. Exception is the contribution from the outer surface of the outermost element, which vanishes by virtue of other arguments.

and take the opportunity to verify our approach against the commercial software COMSOL[9] and to check the influence of the two different approaches in treating the boundary.

We first compare the two different implementations for three different sizes (radii): 2, 2.5, and 3 nm[10]. The results are shown in Figure S7. It is clear that two separate regimes exist: below and beyond the critical frequency. Below, at the dipole frequency the two approaches agree very well in both the amplitude and the position of the resonance (maximum difference of 3.62% in amplitude for the largest sphere, and 0.31% for the position for the 2.5 nm one). Beyond the critical frequency, the two approaches disagree with respect to the position of the (two) Bennett resonances. In light of the discussions of [S2], [S17], [S18] and ours above, we argue that the disagreement, owing to (what should be) a harmless difference in perspective in the boundary conditions, originates from the extreme sensitivity of the spectrum beyond the critical frequency on the computational domain. The two approaches are not well-converged and as such *do not need* to agree. Besides, both results (again, beyond the critical frequency) are not meaningful.

As an experiment, in order to complete the verification of our approach, we implement the stricter boundary condition $\mathbf{P} = \mathbf{0}$ and compare the two solution strategies once more. We include here a simulation for the sphere of radius 2 nm (as before, 2.0003 nm), where the spill-out allowance has been increased to $35a_0$ (see Figure S8). As expected from our previous discussion, we observe only slight changes in the dipole resonance and a whole array of Bennett resonances. The increase in s leads to a better agreement between our approach when the relaxed boundary conditions are used and COMSOL (compare the red line with the blue dots) when it comes to the first Bennett resonance. This is expected: As s is increased, both approaches converge better to the physical solution (only achievable in the infinite-s limit) where the discrete Bennett resonances collapse into a shoulder [S17]; this starts to be visible in this simulation. Most striking, however, is the comparison between COMSOL and our approach where the stricter boundary condition is used (compare the red line with the black dots). The agreement across the entire spectrum is excellent; we still caution however that the agreement beyond the critical frequency is essentially *meaningless* from a physical viewpoint. We quantify our statements in Table S2.

[9] The authors of [S17] have (commendably!) made their implementation public; we use their implementation as black box, modifying it in order to achieve congruence between inputs.

[10] More precisely, 2.0003, 2.5003, and 3.0001 nm, since in the COMSOL implementation the radius is constructed from the number of electrons in the jellium sphere.

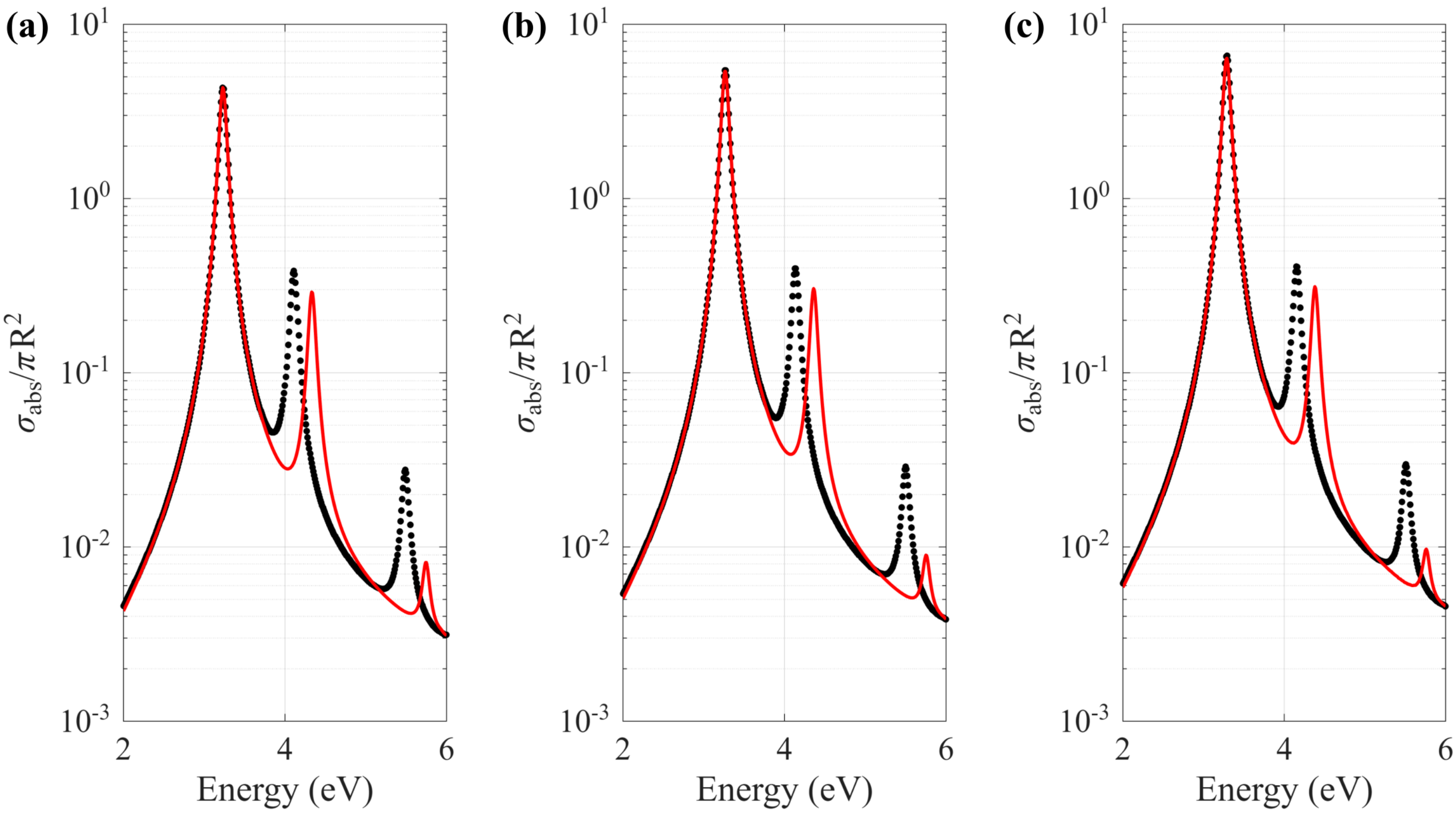

Figure S7: Comparison of COMSOL (red line) with our VIE approach (black dots). The absorption cross section is at (a) 2 nm, (b) 2.5 nm, and 3.0 nm.

Table S2: Relative error in the energy of the resonances that appear in Figure S8 between COMSOL and our approaches using the two perspectives in the boundary condition we detailed previously. The resonances are enumerated from lower to higher; the sixth resonance from the relaxed VIE method does not have a counterpart in the other two approaches and we exclude it below.

Resonance number	Error (%) between COMSOL and relaxed VIE method	Error (%) between COMSOL and strict VIE method
1	0.31	0.31
2	0.28	0.00
3	1.49	0.00
4	2.81	0.00
5	3.35	0.00

S12 Simulation parameters for OpenSANS

In this Section, we include in Table S3 the full simulation details behind Figure 2 of the main text and concerning the software OpenSANS [S7]. The wavelengths below translate to a range of $(2.48, 8.27)$ eV. Since the field is

Table S3: Simulation setup for OpenSANS and for the results presented in Figure 2 of the main text. The LRA-relevant parameters are shown in the left table, while the HDM-relevant ones in the right.

Simulation Parameter	Input Value
Number of wavelengths	301
Range of wavelengths	$[150, 500]$ nm
$l_{\max}$	2
θ_{inc}	0 rad
ϕ_{inc}	0 rad
Polarization	Transverse Magnetic
ω_p	6.04 eV [S19]
γ	0.1 eV [S2]

Simulation Parameter	Input Value
Number of wavelengths	1001
Range of wavelengths	$[150, 500]$ nm
$l_{\max}$	2
θ_{inc}	0 rad
ϕ_{inc}	0 rad
Polarization	Transverse Magnetic
ω_p	6.04 eV [S19]
γ	0.1 eV [S2]
υ_F	0.82×10^6 m/s [S3]

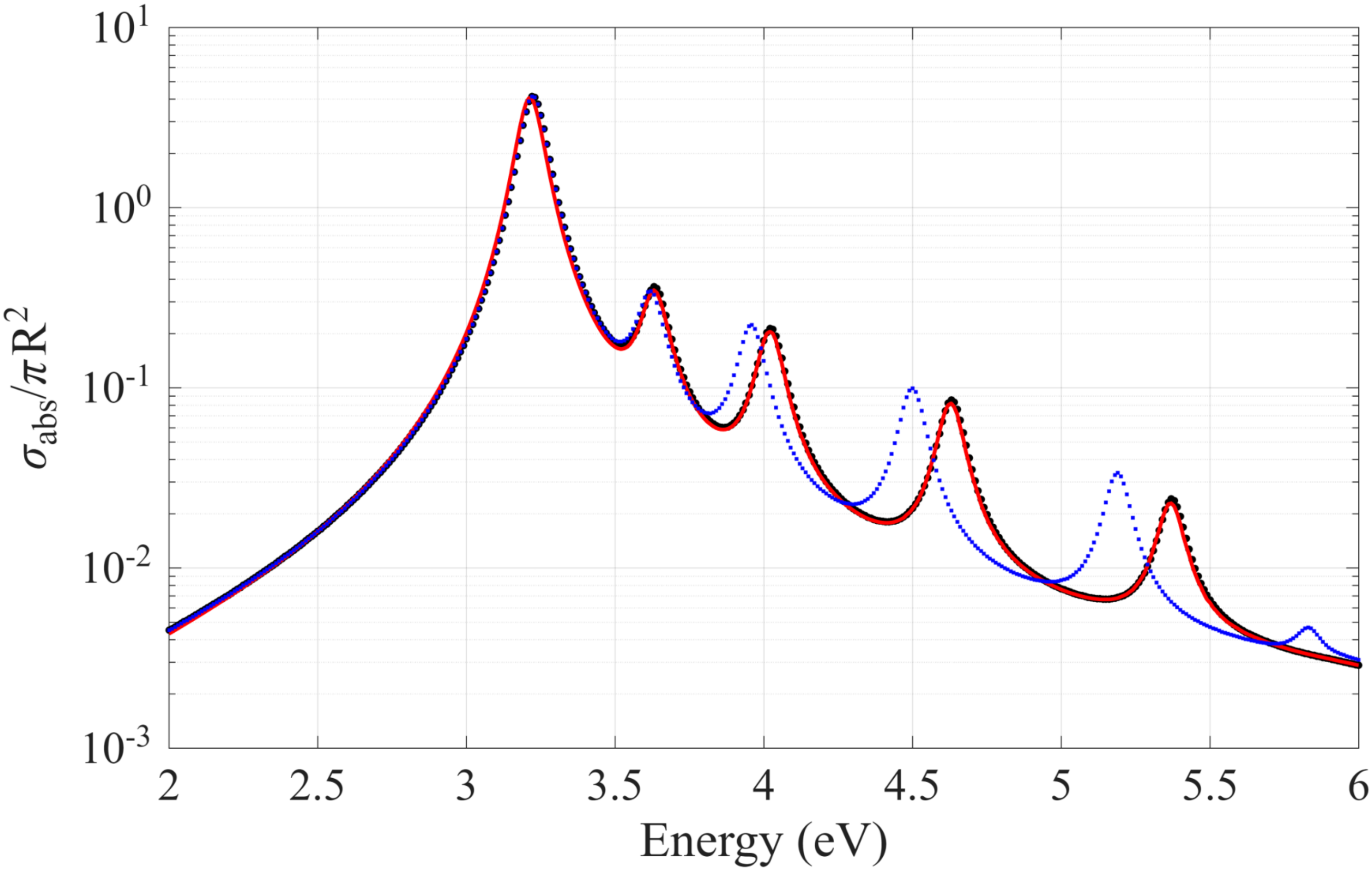

Figure S8: Comparison of the normalized absorption cross section obtained from COMSOL with two VIE implementations. COMSOL results correspond to the red line, the VIE method using the relaxed boundary conditions corresponds to the blue dots, and the VIE method using the strict boundary conditions corresponds to the black dots.

specified as TM, this means that its magnetic field is fully described by the $\mathbf{M}$ planar vector wave function. In spherical coordinates, this translates to a polarization of $\hat{\boldsymbol{\theta}}$, and under $(\theta_{\text{inc}}, \phi_{\text{inc}}) = (0, 0)$ rad, to $\hat{\mathbf{x}}$. The direction of incidence is defined by $\hat{\mathbf{r}} = \hat{\mathbf{z}}$ if $(\theta_{\text{inc}}, \phi_{\text{inc}}) = (0, 0)$ rad. To calculate the scattering and the absorption cross sections, two different observation meshes are used. They both discretize spherical surfaces and their features are gathered in Table S4.

Table S4: Observation mesh details used for the calculation of scattering and absorption cross sections of Figure 2 of the main text and for OpenSANS. R is the radius of the actual nanosphere that is simulated.

Details	Mesh for σ_{sca}	Mesh for σ_{abs}
Type of mesh elements	triangles	triangles
Number of elements	508	1126
Number of vertices	256	565
Radius	1 m	$1.1R$
Fields calculated at	centroid	incenter

S13 Simulation parameters for the VIE implementation

As before, we collect here the full setup we used for the extraction of data corresponding to Figure 2 of the main text.

We clarify here, and this is relevant also to the more demanding SC-HDM simulations, that we have not performed optimization of the parameters shown above (referring to the ones relevant to computational performance, e.g., number of elements, order of integration). For example, for LRA, we have confirmed that cubic basis

Table S5: Simulation setup for the VIE implementation and for the results presented in Figure 2 of the main text. The LRA-relevant parameters are shown in the left table, while the HDM-relevant ones in the right.

Simulation Parameter	Input Value
Number of wavelengths	301
Range of wavelengths	$[150, 500]$ nm
$l_{\max}$	2
θ_{inc}	0 rad
ϕ_{inc}	0 rad
Polarization	Transverse Magnetic
ω_p	6.04 eV [S19]
γ	0.1 eV [S2]
Order of quadrature	10
Order of the basis functions	cubic
Number of geometric elements	99
Number of computational nodes	298

Simulation Parameter	Input Value
Number of wavelengths	301
Range of wavelengths	$[150, 500]$ nm
$l_{\max}$	2
θ_{inc}	0 rad
ϕ_{inc}	0 rad
Polarization	Transverse Magnetic
ω_p	6.04 eV [S19]
γ	0.1 eV [S2]
υ_F	0.82×10^6 m/s [S3]
Order of quadrature	10
Order of the basis functions	cubic
Number of geometric elements	99
Number of computational nodes	298

functions are not necessary (which we intuitively expect from the absence of derivatives from the formalism) and well-converged results can be retrieved by a minuscule amount of elements/computational nodes. This is enabled by the 1D discretization, as for the tiny (< 5 nm) nanoparticles studied here, only a small amount of elements is necessary to resolve the subnanometric length scale defined by the Fermi wavelength (for sodium $\lambda_F = 0.68$ nm [S20]).

Concerning the mapping meshes, these are very similar to the ones OpenSANS is using. We collect them in Table S6. We note that the calculation of the absorption cross section was very sensitive to the mesh used, rendering imperative to use the same mesh in both implementations.

Table S6: Observation mesh details used for the calculation of scattering and absorption cross-sections of Figure 2 of the main text and for the VIE implementation. R is the radius of the actual nanosphere that is simulated.

Details	Mesh for σ_{sca}	Mesh for σ_{abs}
Type of mesh elements	triangles	triangles
Number of elements	4232	1126
Number of vertices	2118	565
Radius	1 m	$1.1R$
Fields calculated at	incenter	incenter

The data corresponding to Figure 3 of the main text and for the LRA and HDM simulations are shown in Table S7, while for the SC-HDM in Table S8. The sphere has a radius of 2 nm. The mapping meshes concern here not

Table S7: Simulation setup for the VIE implementation and for the results presented in Figure 3 of the main text. The LRA-relevant parameters are shown in the left table, while the HDM-relevant ones in the right.

Simulation Parameter	Input Value
Number of wavelengths	301
Range of wavelengths	$[2, 5]$ eV
$l_{\max}$	2
θ_{inc}	0 rad
ϕ_{inc}	0 rad
Polarization	Transverse Magnetic
ω_p	6.04 eV [S19]
γ	0.1 eV [S2]
Order of quadrature	10
Order of the basis functions	cubic
Number of geometric elements	99
Number of computational nodes	298

Simulation Parameter	Input Value
Number of wavelengths	301
Range of wavelengths	$[2, 5]$ eV
$l_{\max}$	2
θ_{inc}	0 rad
ϕ_{inc}	0 rad
Polarization	Transverse Magnetic
ω_p	6.04 eV [S19]
γ	0.1 eV [S2]
υ_F	0.82×10^6 m/s [S3]
Order of quadrature	10
Order of the basis functions	cubic
Number of geometric elements	99
Number of computational nodes	298

Table S8: Simulation setup our VIE method for Figure 3 of the main text for the SC-HDM.

Simulation Parameter	Input Value
Number of energies	301
Range of energies	$[2, 5]$ eV
$l_{\max}$	2
θ_{inc}	0 rad
ϕ_{inc}	0 rad
Polarization	Transverse Magnetic
ω_p	6.04 eV [S19]
γ	0.1 eV [S2]
η	1 [S2]
a_n	$2a_0/1.05$
s	$15a_0$
Order of quadrature	10
Order of the basis functions	cubic
Number of geometric elements	100
Number of computational nodes	301

only the ones needed for the calculation of far and near fields (and by extension cross sections) but also for the induced charge density. For this quantity the meshes are circular disks in the xz plane. Their radius depends on the hydrodynamic model used. For HDM they have the same radius as the nanosphere (as the induced charge density vanishes at the interface of the sphere and beyond). For SC-HDM it extends beyond the sphere to graphically depict the spill-out. The details of the meshes are shown below in Table S9.

Table S9: Observation mesh details used for the calculation of scattering and absorption cross-sections and of the induced charge density of Figure 3 of the main text and for the VIE implementation. R is the radius of the actual nanosphere that is simulated. The induced charge density is $\rho_1 = en_1$. If SC-HDM is used, then the radius of the mesh for σ_{abs} is $1.1(R + s)$.

Details	Mesh for σ_{sca}	Mesh for σ_{abs}	Mesh for ρ_1 (HDM)	Mesh for ρ_1 (SC-HDM)	Mesh for $d_\perp$
Type of mesh elements	triangles	triangles	triangles	triangles	line segments
Number of elements	4232	1126	28994	18830	10000
Number of vertices	2118	565	14697	9505	10001
Radius/length	1 m	$1.1R$	R	$R + s$	$R + s$
Fields calculated at	incenter	incenter	incenter	incenter	midpoint

We turn our attention to the data behind Figure S5, where SC-HDM is used and collect the list of inputs in Table S10. For data relevant to the absorption cross section, see Table S6. We use the same data for Figure S7,

Table S10: Simulation setup our VIE method for Figure S5. The sphere has a radius of 2 nm.

Simulation Parameter	Input Value
Number of energies	401
Range of energies	$[2, 6]$ eV
$l_{\max}$	2
θ_{inc}	0 rad
ϕ_{inc}	0 rad
Polarization	Transverse Magnetic
ω_p	5.90 eV [S21]
γ	0.1 eV [S2]
η	1 [S2]
a_n	$2a_0/1.05$
s	$10a_0$ to $60a_0$ with a step of $5a_0$
Order of quadrature	10
Order of the basis functions	cubic
Number of geometric elements	100
Number of computational nodes	301

but set $s = 15a_0$. For Figure S8, s is increased to $35a_0$.

S14 Computational resources and implementation

All calculations for the LRA, HDM, and SC-HDM with OpenSANS or our VIE method have been carried out on a personal laptop equipped with an 8/16 AMD Ryzen 7 5800H CPU and 16 GB RAM. COMSOL simulations were performed on a workstation equipped with a 24/48 AMD Ryzen Threadripper 3960X CPU and 128 GB RAM.

To facilitate the realization of our VIE approach, we include the main computational engine (written in MATLAB) together with some introductory demos. We clarify that the code shared is undocumented and not written in an educational manner. We aspire to extend it for other canonical geometries, rewrite it and share it as a standalone software. The preliminary code can be found here.

References

[S1] N. A. Mortensen, P. A. D. Gonçalves, F. A. Shuklin, J. D. Cox, C. Tserkezis, M. Ichikawa, C. Wolff, *Nanophotonics* **2021**, *10*, 14 3647.

[S2] C. Ciracì, F. Della Sala, *Phys. Rev. B* **2016**, *93* 205405.

[S3] G. Toscano, J. Straubel, A. Kwiatkowski, C. Rockstuhl, F. Evers, H. Xu, N. A. Mortensen, M. Wubs, *Nat. Commun.* **2015**, *6*, 1 7132.

[S4] W. Yang, *Phys. Rev. A* **1986**, *34* 4575.

[S5] G. S. Ho, V. L. Lignères, E. A. Carter, *Comput. Phys. Commun.* **2008**, *179*, 11 839.

[S6] J. P. Perdew, A. Zunger, *Phys. Rev. B* **1981**, *23* 5048.

[S7] C. Mystilidis, X. Zheng, G. A. E. Vandenbosch, *Comput. Phys. Commun.* **2023**, *284* 108609.

[S8] C. F. Bohren, D. R. Huffman, *Absorption and Scattering of Light by Small Particles*, Wiley, New York, **1998**.

[S9] W. C. Chew, *Waves and Fields in Inhomogenous Media*, Wiley-IEEE Press, **1995**.

[S10] J.-M. Jin, *The Finite Element Method in Electromagnetics*, Wiley-IEEE Press, **2014**.

[S11] N. M. Temme, Interpolation, https://dlmf.nist.gov/3.3, **2025**.

[S12] T. M. Dunster, Legendre and related functions, https://dlmf.nist.gov/14.30, **2025**.

[S13] H. Feshbach, P. M. Morse, *Methods of Theoretical Physics*, McGraw-Hill, New York, **1953**.

[S14] F. W. J. Olver, L. C. Maximon, Bessel functions, https://dlmf.nist.gov/10.51, **2025**.

[S15] G. von Winckel, Legendre-gauss quadrature weights and nodes, MATLAB Central File Exchange, **2004**, URL https://nl.mathworks.com/matlabcentral/fileexchange/4540-legendre-gauss-quadrature-weights-and-nodes.

[S16] W. C. Gibson, *The Method of Moments in Electromagnetics*, Chapman & Hall/CRC, Boca Raton, FL, 2nd edition, **2008**.

[S17] H. M. Baghramyan, F. Della Sala, C. Ciracì, *Phys. Rev. X* **2021**, *11* 011049.

[S18] W. Yan, *Phys. Rev. B* **2015**, *91* 115416.

[S19] S. Raza, S. I. Bozhevolnyi, M. Wubs, N. Asger Mortensen, *J. Phys.: Condens. Matter* **2015**, *27*, 18 183204.

[S20] N. W. Ashcroft, N. D. Mermin, *Solid State Physics*, Harcourt College Publishers, **1976**.

[S21] C. Xia, C. Yin, V. V. Kresin, *Phys. Rev. Lett.* **2009**, *102* 156802.
